# Comprehensive comparative-genomic analysis of Type 2 toxin-antitoxin systems and related mobile stress response systems in prokaryotes

**DOI:** 10.1186/1745-6150-4-19

**Published:** 2009-06-03

**Authors:** Kira S Makarova, Yuri I Wolf, Eugene V Koonin

**Affiliations:** 1 National Center for Biotechnology Information, NLM, National Institutes of Health, Bethesda, Maryland 20894, USA

## Abstract

**Background:**

The prokaryotic toxin-antitoxin systems (TAS, also referred to as TA loci) are widespread, mobile two-gene modules that can be viewed as selfish genetic elements because they evolved mechanisms to become addictive for replicons and cells in which they reside, but also possess "normal" cellular functions in various forms of stress response and management of prokaryotic population. Several distinct TAS of type 1, where the toxin is a protein and the antitoxin is an antisense RNA, and numerous, unrelated TAS of type 2, in which both the toxin and the antitoxin are proteins, have been experimentally characterized, and it is suspected that many more remain to be identified.

**Results:**

We report a comprehensive comparative-genomic analysis of Type 2 toxin-antitoxin systems in prokaryotes. Using sensitive methods for distant sequence similarity search, genome context analysis and a new approach for the identification of mobile two-component systems, we identified numerous, previously unnoticed protein families that are homologous to toxins and antitoxins of known type 2 TAS. In addition, we predict 12 new families of toxins and 13 families of antitoxins, and also, predict a TAS or TAS-like activity for several gene modules that were not previously suspected to function in that capacity. In particular, we present indications that the two-gene module that encodes a minimal nucleotidyl transferase and the accompanying HEPN protein, and is extremely abundant in many archaea and bacteria, especially, thermophiles might comprise a novel TAS. We present a survey of previously known and newly predicted TAS in 750 complete genomes of archaea and bacteria, quantitatively demonstrate the exceptional mobility of the TAS, and explore the network of toxin-antitoxin pairings that combines plasticity with selectivity.

**Conclusion:**

The defining properties of the TAS, namely, the typically small size of the toxin and antitoxin genes, fast evolution, and extensive horizontal mobility, make the task of comprehensive identification of these systems particularly challenging. However, these same properties can be exploited to develop context-based computational approaches which, combined with exhaustive analysis of subtle sequence similarities were employed in this work to substantially expand the current collection of TAS by predicting both previously unnoticed, derived versions of known toxins and antitoxins, and putative novel TAS-like systems. In a broader context, the TAS belong to the resistome domain of the prokaryotic mobilome which includes partially selfish, addictive gene cassettes involved in various aspects of stress response and organized under the same general principles as the TAS. The "selfish altruism", or "responsible selfishness", of TAS-like systems appears to be a defining feature of the resistome and an important characteristic of the entire prokaryotic pan-genome given that in the prokaryotic world the mobilome and the "stable" chromosomes form a dynamic continuum.

**Reviewers:**

This paper was reviewed by Kenn Gerdes (nominated by Arcady Mushegian), Daniel Haft, Arcady Mushegian, and Andrei Osterman. For full reviews, go to the Reviewers' Reports section.

## Background

Bacterial toxin-antitoxin systems (TAS, also referred to as TA loci) originally have been characterized in the 1980s as molecular systems encoded in plasmids and ensuring the persistence of a plasmid in a host lineage during replication by making the cells "addicted" to the plasmid so that only plasmid-containing daughter bacteria survived after a cell division [1,2]. As implied by their name, the overwhelming majority of TAS consist of two components encoded in an operon [3]. The toxin component of all TAS is a protein that kills cells if expressed above a certain level, whereas the antitoxin component regulates the expression of the toxin and/or inactivates the toxin, thereby preventing cell killing. The mechanism of post-segregational killing of plasmid-less cells, which is also the mechanism of plasmid maintenance, is simple and elegant. The antitoxin is metabolically unstable unless in a complex with the toxin, whereas the toxin is considerably more stable. Therefore, unless the antitoxin is continuously replenished through gene expression, the free toxin accumulates in amounts sufficient to kill a cell, which is what occurs after cell division if a daughter cell does not receive the TAS-encoding plasmid [3-5]

The TAS are currently classified into two major types on the basis of the nature of the antitoxin [3,6]. Type I TAS encompass an antisense RNA antitoxin that is complementary to the toxin mRNA and prevents its translation; the toxin of type I TAS is, typically, a small hydrophobic protein with a holin-like mechanism of action that kills cells by impairing the membrane [6,7].

Type II TAS employ a protein antitoxin to keep the toxin inactivated via protein-protein interaction. These systems show considerable structural and functional diversity among both the toxins and the antitoxins. The most common activity of Type II toxins seems to be that of an mRNA-specific endonuclease, termed interferases [8,9]. In particular, the interferase activity was demonstrated for the widespread toxins of the RelE and MazF. In addition, there are at least two other mechanisms of action of Type II toxins. The well-explored CcdB and ParE toxins are inhibitors of DNA gyrase that abrogate cell reproduction by blocking DNA replication. The recently characterized HipA toxin is a protein kinase [10] that abrogates bacterial reproduction and renders bacterial cells dormant by inhibiting translation through phosphorylation of the elongation factor EF-Tu [11]. All type II antitoxins are dual-function, two-domain proteins that consist of a protein-protein interaction domain and a DNA-binding domain. When not complexed with other proteins, antitoxins have largely disordered structures and are highly susceptible to proteolysis, and hence unstable. Upon interaction with the respective toxins via their protein-protein interaction domains, the antitoxins assume compact structures and are accordingly stabilized. The antitoxin binding inhibits the activity of the toxin, and the stable TA complex binds to the operator of the corresponding TAS operon via the DNA-binding domain of the antitoxin and (auto)represses its transcription. Thus, the antitoxin exerts control over the activity of the TAS at two levels, by directly inhibiting the toxin and by repressing the expression of both TAS components.

The TAS were originally discovered on plasmids and appeared to be devices employed by the plasmid replicons to "manipulate" the host bacteria to maintain the plasmids. However, when multiple bacterial and archaeal genomes were sequenced, it became obvious that many of them contained multiple, diverse TA loci [3-5,12,13]. Concomitantly, functions of TAS in bacterial physiology were discovered that seem to have nothing to do with plasmid maintenance, namely, a central role in stress response. The best characterized model is the involvement of the RelBE TAS in bacterial stringent response to amino acid starvation. During starvation, the RelE toxin is activated as a result of the proteolysis of the RelB antitoxin which also leads to the activation of transcription of the *relBE *operon. The end result is the extensive cleavage of ribosome-associated mRNA, a major shutdown of translation, and concomitant increase of the pool of charged tRNAs [14]. It is thought that this modulation of the state of the translation system results in the adjustment of nutrient consumption and to increased translation fidelity, two critical adaptations that apparently allow bacteria to survive starvation. In other words, the RelBE TAS seems to exert quality control of protein synthesis. Similar observations were reported for the MazEF TAS where MazF is a ribosome-independent mRNA interferase [15].

The study of MazEF led to a major reappraisal of the biological effects of TAS. Originally, it was proposed that the unleash of MazF under stress induced programmed cell death (PCD) in *E. coli *[16]. Although TAS-mediated PCD might indeed occur under a variety of stress conditions [17,18], the more common mechanism of TAS seems to be the induction of reversible bacteriostasis (dormancy or persistence) [19]. The induction of persistence is also the mechanism of action of the HipAB TAS that was recently reported to be mediated by translation inhibition via phosphorylation of EF-Tu [11]. On the other hand, a recent study on the developmentally complex bacterium *Myxococcus xanthus *showed that the solitary MazF toxin (mRNA interferase) triggered PCD which in this case is a regular developmental stage [20]. Thus, in addition to their well-characterized role in stress response, some of the TAS components can be prokaryotic development regulators. Generally, it appears that, depending on the specific conditions, TAS can affect the fates of prokaryotic cells in different manners. In particular, TAS-mediated persistence and PCD are likely to be a common mechanism of resistance to various environmental assaults including diverse antibiotics and other drugs [21,22].

The results briefly summarized above suggest that TAS are essential components of prokaryotic cell biology rather than simply plasmid addiction modules. It might be premature to consider this concept firmly established, and the proposed cellular functions of TAS remain a matter of debate [23,24]. Indeed, deletion of all 5 TA loci in E. coli did not result in a significant decrease of the fitness of the bacteria [23]. Thus, the possibility is still considered that the TA operons are completely selfish and are maintained in the population owing to the segregational (recombinational) bias for addictive modules [5,24]. A potential compromise between a purely selfish life style of TAS and integral cellular functions could be a role of chromosomally encoded TAS in the protection of prokaryotic cells against post-segregational killing induced by plasmid-encoded homologous TAS whereby the antitoxin encoded by a chromosomal gene sequesters a plasmid-encoded toxin. Experimental evidence of such protection was reported, and elimination of the chromosomal TAS in the presence of the respective plasmid did adversely affect the fitness of the host bacterium [25].

Despite the uncertainty about "normal" functions of TAS or, perhaps, fueled by the multiplicity of possibilities in this area, the increasing interest in TAS resulted in both experimental [3,4,6] and computational [13,26] identification and prediction of scores of new TA modules in most of the sequenced prokaryotic genomes, with the exception of small genomes of parasitic bacteria, especially, intracellular parasites. Comparative-genomic studies also demonstrated a remarkable horizontal mobility of TA operons [13,26]. Identification and annotation of genes for toxins and antitoxins are problematic due to the small size of most of these genes and likely atypical (because of frequent lateral transfers) GC content and codon usage. Recently, these problems prompted the development of specialized software for the identification of TA gene pairs [27,28]. These tools utilize the information on already characterized families of toxins and antitoxins and are helpful, primarily, for finding missing ORFs in two-gene TA operons.

The steadily increasing diversity of TAS paralleled by the exponential growth of number of sequenced genomes suggests that numerous new TAS await discovery in genomic sequences that are already present in current databases. Here we attempt to identify new TAS (and highly derived versions of the known ones) by using comparative-genomic approaches guided by the "guilt by association" principle [29-31] and specific properties of TAS. We also present a comprehensive survey of TAS in the sequenced archaeal and bacterial genomes, in an attempt to reveal general trends in their distribution and evolution.

## Results and discussion

### Two complementary approaches for the prediction of new TAS

**Figure 1:**
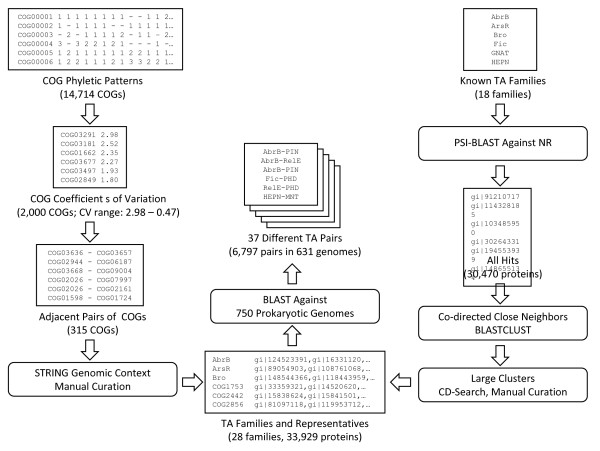
**Two computational strategies for the identification of TAS**.

The TAS are prone to frequent horizontal gene transfer (HGT) and intragenomic recombination, so they are commonly described as mobile genetic elements [26,27,32]. Apparently, as a result of this mobility, TAS show a patchy distribution among prokaryotic genomes, with some genomes encoding tens of TAS and others encoding only a few or none. Keeping in mind this characteristic distribution of TAS across the prokaryotic world, we utilized phyletic patterns of COGs (Clusters of Orthologous Genes [33]) to develop a search strategy for pairs of genes that are significantly non-uniformly distributed among prokaryotic genomes and, in addition, form recurrent two-gene (predicted) operons (Figure 1).

For each COG from 110 bacterial and archaeal genomes (see Methods for details), the variability of the abundance of the member genes was estimated. To this end, the coefficient of variation (CV) was computed as the ratio of the standard deviation of the number of paralogs to the mean number of paralogs, excluding species that had no genes from the given COG. The representatives of 2000 COGs with the highest CV values (ranging from 2.98 to 0.47) were mapped to the genome DNA, and pairs of COGs that were adjacent at least three times in at least one genome were selected for further analysis. This filter yielded 315 pairs of COGs (Additional File 1) which were examined case by case using the STRING program [34] to exclude those that belonged to longer (three or more genes) conserved operons.

The 23 pairs of COGs that passed the final filter are listed in Table 1. As expected, the majority of the identified COGs were components of already characterized TAS. Notably, this group included the *hicA-hicB *gene pair that has been predicted to comprise a new TAS pair on the basis protein sequence and comparative-genomic analysis [32], a prediction that was supported by a recent experimental study [35] (see below). Several of these gene pairs were not so far associated with any known TAS, and deserve further attention as potential candidates for new TAS. Three of these candidate TAS consist of distinct subfamilies of minimal nucleotidyltransferases (MNT) and the accompanying subfamilies of the HEPN (Higher Eukaryotes and Prokaryotes Nucleotide-binding) proteins. Both these protein families have been described previously [36-38] but their biological functions remain elusive.

**Table 1:** Previously characterized and new candidate TAS detected with the the first approach

**COG number**	**Toxin family**	**CV**	**COG number**	**Antitoxin family**	**CV**
COG1848	PIN	1.1	COG8614	RHH	1.0

**COG3832**	**Aha1 family**	1.2	COG0640	ArsR family HTH	0.8

**COG1708**	**MNT**	0.9	**COG2250**	**HEPN**	1.0

COG4679	RelE	0.8	COG5606	HTH	0.6

COG2026	RelE	0.7	**COG7997**	**MJ1172-like**	0.7

COG3668	RelE	0.9	COG2161	StbD/axe	0.5

**COG1708**	**MNT**	0.4	**COG2445**	**HEPN**	0.9

COG3657	RelE	0.7	COG3636	HTH	0.6

COG1848	PIN	0.4	COG2002	AbrB/MazE/PemI	0.9

COG3668	RelE	0.9	COG3609	RHH	0.4

**COG1669**	**MNT**	**0.7**	**COG2361**	**HEPN**	0.6

COG9434	MazF	0.6	COG5302	CcdA	0.7

COG1598	hicB	0.7	COG1724	hicA	0.6

COG3549	RelE	0.7	COG3093	HTH	0.6

COG2026	RelE	0.4	COG2161	StbD_axe	0.9

COG3668	RelE	0.7	COG9004	RHH	0.5

COG6187	RelE	0.6	COG2944	HTH	0.6

COG1487	PIN	0.7	COG4710	RHH	0.4

COG1487	PIN	0.6	COG4456	AbrB/MazE/PemI	0.4

COG3742	PIN	0.5	COG4423	RHH	0.4

COG1487	PIN	n/a	COG5450	RHH	0.5

COG numbers below 5600 correspond to the COGs that are available on the NCBI site:

COGs that were predicted to include novel toxins and antitoxins in this work are shown by bold type.

CV – coefficient of variation.

In addition, we employed a more straightforward and widely used approach to detect potential new TAS components (Figure 1). It was observed previously that a particular toxin can combine in (predicted) TAS operons with different, often structurally unrelated antitoxins, and conversely, homologous antitoxins combine with different toxins [3,4,12]. Therefore, a detailed analysis of the gene neighbors of known or predicted TAS-related genes has the potential to uncover previously unnoticed TAS components (see Methods for details). To this end, we first used representatives of all known TA protein families and the new candidates identified with the first approach as queries for exhaustive PSI-BLAST [39] searches. To characterize the diversity of each family as completely as possible, the most divergent sequences detected in these searches were also used as queries for a second round of PSI-BLAST searches. Altogether, for 18 superfamilies of (predicted) toxins and antitoxins, approximately 30,470 hits were collected for further analysis. All these genes were mapped onto the respective chromosome or plasmid sequences, and co-directed neighbors separated by less than 100 base pairs. The protein sequences thus obtained were classified into families using the CDD database [40] and/or BLASTCLUST (see Methods for details), and all the clusters for which at least 20 instantiations were detected (78 distinct pairs) were examined case by case (Table 2).

**Table 2:** Previously characterized and potential new TAS components detected with the second approach

**Query family**	**Source**	**Adjacent gene family**	**Adjacent gene function; reasons if discarded**.	**Number of occurrences observed**
**AbrB/MazE**	[3]	PIN	Nuclease	312
		
		MazF	RNA interferase	97
		
		Fic/Doc	AMPylation enzyme	48
		
		RelE	RNA interferase	27

**ArsR**	Table 1	**COG3832**	Aha1 domain	280
		
		COG0394*	Arsenate reductase arsC; Part of a larger conserved gene associations	98
		
		COG2217*	Cation transport ATPase; Part of a larger conserved gene associations	83
		
		COG0798*	Arsenite efflux pump ACR3; Part of a larger conserved gene associations	74
		
		COG2391*	YeeE/YedE family, DUF395; Part of a larger conserved gene associations	64
		
		COG1055*	Arsenical pump membrane protein; Part of a larger conserved gene associations	53

**RHH**	[3]	RelE	RNA interferase	376
		
		PIN	Nuclease	335
		
		**GNAT**	Acetyltransferase	139
		
		MazF	RNA interferase	62
		
		COG3505*	TraG/TraD/VirD4 family; Part of a larger conserved gene associations	58
		
		**COG2929**	DUF497	55
		
		ParA*	ParA, plasmid partitioning ATPase; Part of a larger conserved gene associations	54
		
		HicA	RNA interferase	26
		
		RHH	DNA-binding domain	23
		
		COG0716*	Flavodoxin; Part of a larger conserved gene associations	22
		
		COG4962*	Type II/IV secretion system protein; Part of a larger conserved gene associations	21

**Fic_Doc**	[3]	AbrB	DNA-binding domain	55
		
		xre	DNA-binding domain	22
		
		**yhfG**	Unknown	11

**MazF**	[3]	AbrB	DNA-binding domain	107
		
		RHH	DNA-binding domain	81
		
		MazF/ccd	RNA interferase	43
		
		**XF1863**	Unknown	29

**RelE**	[3]	xre	Transcriptional regulator	730
		
		RHH	DNA-binding domain	510
		
		PHD	DNA-binding domain	337
		
		**COG2856**	Zn peptidase (fused to HTH)	15
		
		**COG1753**	Predicted DNA-binding domain; RHH fold	10

**PIN** **RNA nuclease**	[3]	RHH	DNA-binding domain	366
		
		AbrB	DNA-binding domain	348
		
		PHD	DNA-binding domain	285
		
		**COG2442**	Protein of unknown function DUF433	97
		
		**COG2886**	Uncharacterized protein family (UPF0175)	46
		
		**COG2856**	Zn peptidase (fused to HTH)	42
		
		MazF/ccd	COG5302	41
		
		COG1211*	4-diphosphocytidyl-2-methyl-D-erithritol synthase; Part of a larger conserved gene associations	39
		
		**MerR**	Transcriptional regulator	33
		
		COG1066*	Sms; Part of a large conserved gene associations	26
		
		COG1092*	SAM-dependent methyltransferase; Part of a larger conserved gene associations	26
		
		**COG2880**	Predicted DNA-binding protein; AbrB superfamily	24
		
		pfam00155*	Aminotransferase; Part of a larger conserved gene associations	24
		
		COG5257*	Translation initiation factor 2; Part of a larger conserved gene associations	20
		
		**COG1753**	Predicted DNA-binding domain; RHH fold	20

**PHD**	[3]	PIN	Nuclease	276
		
		**SMa0917**	PemK/MazFI	15

**MNT**	Table 1	HEPN	Unknown	445

**HEPN**	Table 1	MNT	Predicted nucleotidyltransferase	482

**Xre**	[3]	RelE	RNA interferase	614
		
		HipA	EF-Tu kinase	244
		
		**COG2856**	Zincin protease	194
		
		xre	A variety of proteins containing xre-like HTH, many fused with various domain, not a distinct set	97
		
		PIN	Nuclease	67
		
		**DUF397**	Unknown	64
		
		COG0800*	2-keto-3-deoxy-6-phosphogluconate aldolase; Part of a larger conserved gene associations	46
		
		COG3842*	PotA is ABC-type transporter; Part of a larger conserved gene associations	40
		
		PA2784-like*	A membrane protein, likely an exporter	36
		
		YoaS-like*	A membrane protein, likely permease	32
		
		**antirepressor**	BRO family; KilA – letal to host cells	30
		
		**GNAT**	Acetyltransferase	30
		
		COG3063*	Tfp pilus assembly protein PilF; Part of a large conserved gene associations	27
		
		PA4076-like*	A membrane protein, likely an exporter	27
		
		COG4974*	Site-specific recombinase XerD; Apparent phage components with another function	25
		
		COG0483*	Archaeal fructose-1,6-bisphosphatase; Part of a larger conserved gene associations	21

**xre COG5642 subfamily**		**COG5654**	Predicted transcriptional regulator	118

**HipA**	[3]	xre	Transcriptional regulator	333
		
		COG3550	HipA C-terminal	51

**COG2856**	[60]	xre	Transcriptional regulator	145
		
		PIN	Nuclease	60
		
		RelE	RNA interferase	22

**COG2880**	[13]	PIN	Nuclease	25

**COG3832**	Table 1	**ArsR**	Transcriptional regulator	259

**COG4636**	[13]	COG4636	Predicted endonuclease	114

**COG4679 (RelE family)**	[13]	**COG5606**	Predicted RNA interferase	43

New TAS discussed in this work are shown in bold type; other associations marked by asterisk in column 3 were disregarded for reasons indicated in column 4.

As with the first approach, most of the pairs belonged to already known TAS, and several pairs were found to belong to larger operons and were, accordingly, discarded (Table 2). Also, we excluded three conserved gene pairs where a membrane protein was associated with a Xre family repressor (antitoxins in several known TAS) because no type II TAS with a membrane component were detected so far, whereas Xre family repressors are known to perform a variety of regulatory functions unrelated to TAS [41-44]. The potential new TAS detected by these complementary approaches are listed in Table 3 and discussed below.

**Table 3:** Predicted new TAS

**Toxin (T)**	**Antitoxin (AT)**	**Comment**
**MNT**	**HEPN**	MNT – minimal nucleotidyltransferase, possible toxin; HEPN – possible substrate binding domain; Structure solved (MNT: 1no5 and HEPN: 1o3u and 1jog). Molecular mechanism unknown.

PIN	**COG2442**	Structure of AT is solved (PDB:2ga1):DNA/RNA-binding 3-helical bundle.

PIN	**COG2880**	Structure of AT is solved (PDB:2nwt); related to AbrB superfamily

PIN	**COG1753**	AT – RHH (RHH); Specific for archaea

PIN	**MerR**	AT: truncated MerR

**COG4679 subfamily**	**COG5606 HTH**	AT – predicted HTH domain; T – predicted RelE superfamily protein

RelE	**MJ1172 RHH**	Specific for methanogens; AT – predicted RHH superfamily protein

MazF	**XF1863**	No prediction for AT

Fic/Doc	**YhfG**	AT – is predicted DNA-binding protein; Specific for enteroproteobacteria

**SMa0917 subfamily**	PHD	T – predicted MazF superfamily protein; Molecular mechanism is likely the same as for MazF toxin

**COG2929**	**COG5304 and COG3514** **Families**	AT: predicted RHH family protein; Molecular mechanism unknown.

**DUF397**	Xre/cro HTH	T – no prediction; molecular mechanism unknown.

**COG2856**	Xre/cro HTH	T – predicted Zn-dependent protease. Often fused to AT domain. Frequent association with RelE and PIN toxins

**COG5654**	**COG5642 subfamily**	AT – xre family HTH; T – RES domain; Molecular mechanism unknown.

**YgiU/MqsR**	Xre/cro HTH	T: motility quorum-sensing regulator mqsR [75]

**GNAT**	Xre/cro HTH	The closest characterized GNAT family acetyltrasferase is involved in antibiotic resistance [88]

**GNAT**	RHH	T – is GNAT family acetyltrasferase

**Bro**	Xre/cro HTH	

**COG3832**	**ArsR-like HTH**	T – Cyclase/dehydratase family protein. (PDB: 1xuv) START domain superfamily

New toxins and antitoxins predicted in this work are shown in bold type

### The HicAB system as a paradigm for the prediction of new TAS

The HicAB system was described in depth previously [32]. Briefly, the evidence in support of the hypothesis that HicAB is a novel TAS is the following: 1) The *hicA *and *hicB *genes form a predicted two-gene operon that is found in many bacterial genomes; 2) both genes encode relatively small proteins; 3) the putative operon shows a strongly non-uniform distribution among the genomes and appears to be mobile, presumably, owing to frequent horizontal transfers and recombination; 4) the HicAB system is also present in many phages and plasmids; 5) one of the proteins, HicB, often contains a helix-turn-helix (HTH) DNA-binding domain of the Xre family or a ribbon-helix-helix (RHH) domains; these domains are often found in experimentally characterized antitoxins; 6) HicB contains a derived RNAse H fold, and HicA contains a double-stranded RNA-binding domain (dsRBD) suggesting that, like with many other TAS, the HicAB system targets RNA. Recently, this prediction was validated by an experimental study which demonstrated that HicA indeed is a nuclease that functions as a translation-independent mRNA interferase, that is, most likely, is a bona fide toxin [35].

Given the successful experimental validation of the prediction, the principal features of the HicAB system listed above and the logic exploited in its analysis were chosen as the paradigm for the prediction and analysis of other TAS as detailed below. Those two-gene models that possessed all of these features could be predicted to function as bona fide TAS with considerable confidence whereas the modules that possessed only some of these characteristics, in particular, the cases where there was no support of the TAS prediction from the domain architecture of the component proteins, could represent other types of stress-response systems

### New antitoxins associated with known toxins

Toxins appear to be more specialized than antitoxins. Most of the toxin protein families (PIN), superfamilies (Kid/CcdB/PemK/MazF) and even folds (Fic/Doc, RelE) seem not to play roles in the cell other than the (broadly defined) toxin function. Thus, the presence of any relatively small protein-coding gene in a conserved two-gene operon that includes a gene for any of the known toxins strongly suggests that the small protein in question is an antitoxin. We identified several such putative new antitoxins as discussed below.

#### The Tad-Ata (RelE-COG5606) system

**Figure 2:**
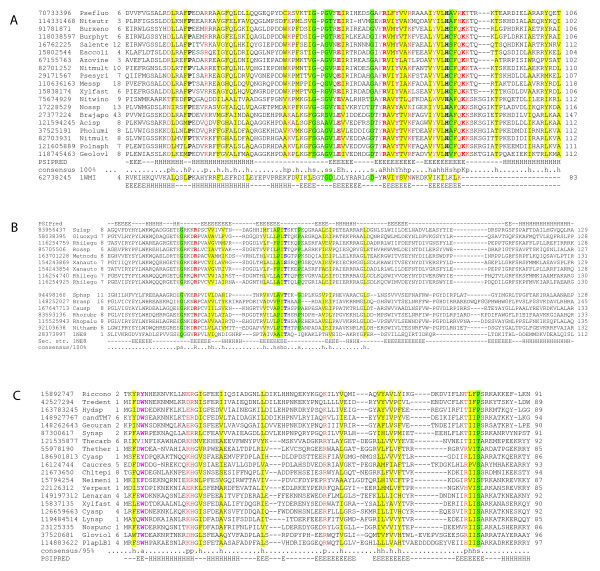
**Predicted new families of toxins**. **A**. Multiple alignment of COG4679 family (RelE interferase supefamily). **B**. Multiple alignment of SMa0917 family (PemK/MazF interferase superfamily). **C**. Multiple alignment of COG2929 family (RelE interferase superfamily) representative. The sequences are denoted by Gene Identification (GI) numbers from the GenBank database and abbreviated species names. Species name abbreviations (generally consisting of 3 first letters of genus name and 4 first letters of species) for all alignments are given in Additional file 13. The positions of the first and the last residues of the aligned region in the corresponding protein are indicated for each sequence. The numbers within the alignment represent poorly conserved inserts that are not shown. The coloring is based on the consensus shown underneath the alignment; h indicates hydrophobic residues (ACFILMVWY), p indicates polar residues (STEDKRNQH), s indicates small residues (AGSVC) and a indicates aromatic residues (WYFH). The secondary structure elements are shown according to structural data if the structure is available or predicted using the PSIPRED program [106]; E indicates β-strand and H indicates α-helix.

A new TAS, denoted Tad-Ata, was recently discovered on *Paracoccus aminophilus *plasmid pAMI2 [45]. The Tad toxins belong to COG4679 (DUF891), a large family of proteins, often referred to as phage-related because they are found in genomes of several bacteriophages and prophages (e.g. gp49 protein in the *E. coli *phage N15). It has been reported that Ata antitoxins belong to COG5606 and COG1396, and that they contain a typical HTH domain of the Xre/Cro family. In the course of this study, we independently made the same observations (Table 3; Additional File 2). In addition, we detected significant sequence similarity between the Tad toxin and the RelE family of RNAses (Figure 2A). For instance, in a PSI-BLAST search initiated with the sequence (gi|17228529) as a query (inclusion threshold 0.01), proteins of the RelE family (e.g. gi|15668242) were detected in the 4^th ^iteration with an E-value of 0.001,). Thus, we propose that, similarly to RelE, the Tad (COG4679) family toxins are mRNA-cleaving RNAses (interferases).

#### New antitoxin families associated with PIN family toxins

The PIN family genes that encode a distinct class of (predicted) RNAse H fold nucleases are abundant in prokaryotic genomes, especially, in archaea [46,47]. We attempted to identify potential novel antitoxins in the neighborhood of PIN genes (Table 2). Here we describe several protein families that are likely to function as previously unnoticed antitoxins for some PIN family nucleases.

**Figure 3:**
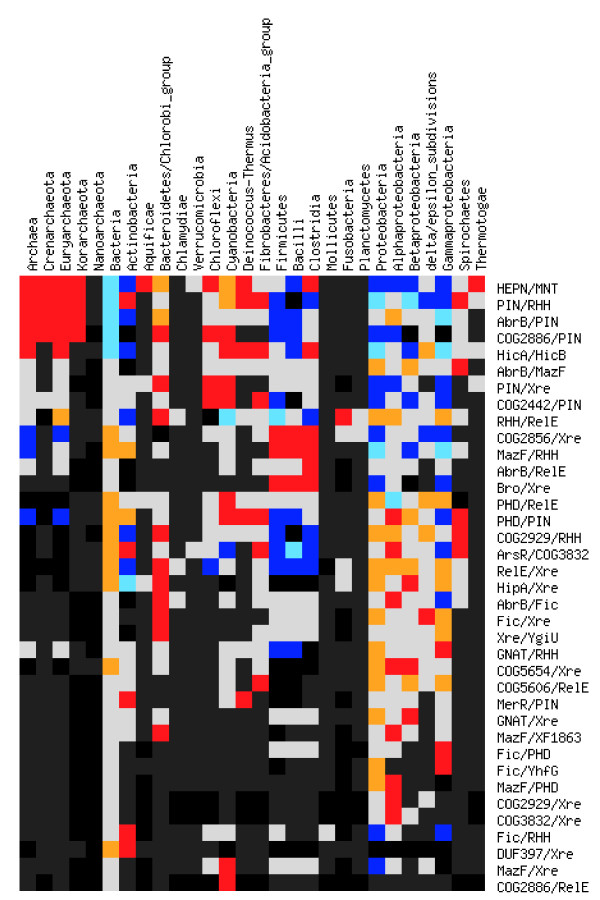
**Distribution of TAS across bacterial and archaeal taxa**. Black: TAS absent in the taxon while random expectation is significantly non-zero. Dark gray: TAS absent in the taxon with random expectation not significantly different from zero. Blue: TAS is significantly underrepresented in a taxon with more than twofold difference from random expectation. Cyan: TAS is significantly underrepresented in a taxon with less than twofold difference from random expectation. Light gray: abundance of a TAS in a taxon does not significantly differ from random expectation. Orange: TAS is significantly overrepresented in a taxon with less than twofold difference from random expectation. Red: TAS is significantly overrepresented in a taxon with more than twofold difference from random expectation. The random expectation estimate is based on the total number of TAS of the given type and the total number of protein-coding genes in the given taxon. The statistical significance was estimated using the χ^2 ^test (critical χ^2 ^value of 3.84 for 1 degree of freedom and p-value of 0.05).

**Figure 4:**
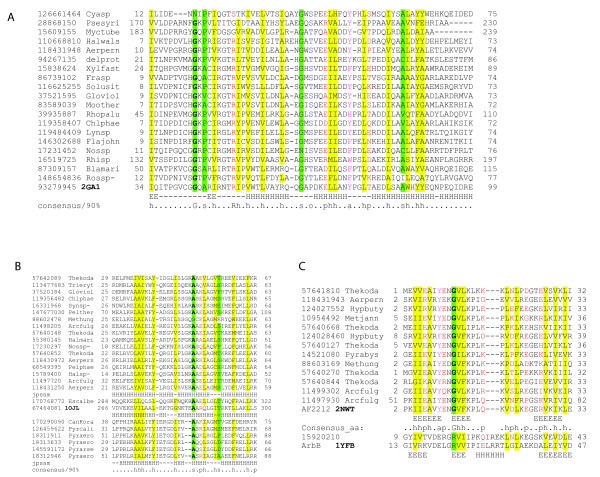
**Predicted new families of antitoxins**. A. Multiple alignment of COG2442 family domain, a predicted DNA-binding antitoxin protein of winged HTH motif superfamily. B. Multiple alignment of COG2886 family of predicted antitoxins containing the HTH domain. C. Multiple alignment of COG2880 family, an AbrB superfamily representative. Designations are the same as in Figure 2.

The **COG2442, DUF433 **family is among the most abundant new PIN-associated predicted antitoxins (Table 2). Proteins of this family are seen in a variety of bacterial and a few archaeal genomes but are most abundant in cyanobacteria (8 PIN-COG2442 pairs in *Anabaena variabilis *ATCC 29413) and chloroflexi (up to 9 pairs in *Roseiflexus *RS1) (Figure 3). The domain length is about 70–80 aa; the structure of one of the proteins from *A. variabilis *has been solved (PDB:2GA1) by Joint Center for Structural Genomics (JCSG) and classified in SCOP as a DNA/RNA-binding 3-helical bundle  (Figure 4A). Hence a clear analogy with other DNA-binding antitoxin proteins that regulate the transcription of the respective toxin-antitoxin operons. A reverse search for conserved gene neighbors of COG2442 did not reveal associations other than that with PIN family genes. However, there are several fusions of the COG2442 domain with DNA-binding winged HTH domains (eg. Rv2018 of *Mycobacterium tuberculosis *H37Rv) most of which are also associated with PIN family genes (e.g. Rv2019) suggesting that the transcription of this TAS could be regulated by more than one mechanism.

**COG2886 **is a family of small proteins associated with PIN genes, mostly, in archaea and cyanobacteria (Figure 3). The PSI-BLAST search (with inclusion threshold 0.01 and almost any query from this COG, for example, gi|118430972) reveals a weak but significant similarity with HTH-domains (eg: gi|170768772 with E-value ~ 0.046; 4^th^–5^th ^iteration), mainly, of the Fis family [48-50]. Multiple alignment and secondary structure prediction support the PSI-BLAST results and reveal a classical HTH motif in the COG2886 family (Figure 4B). There exists a distinct proteins subfamily, paREP6 (*Pyrobaculum aerophilum *repetitive family 6), only distantly related to bona fide COG2886 family members, that is also often associated with PIN-coding gene; secondary structure predictions for this subfamily also support the presence of an HTH motif (Figure 4B). This family is overrepresented in *Pyrobaculum *genomes and is also present in other Thermoproteales and in several other archaea, including the recently sequenced *Korarchaeum cryptofilum *(eg. Kcr_0836, Kcr_0472, Krc_0407 and Krc_0470).

The **COG2880 (DUF104) **family is notable owing to its remarkable expansion in the archaeon *Archaeoglobus fulgidus *(12 paralogs). Some of these genes form clusters and are closely related to each other suggesting tandem duplication that is seen also for the adjacent genes encoding PIN-domain proteins. Recently, the NMR structure of one of the COG2880 proteins from *A. fulgidus *(AF2212) was solved by the Northeast Structural Genomics Consortium (PDB: 2NWT). Searches with the VAST or DALI programs did not detect any structural neighbors with high confidence; however, a PSI-BLAST search (with inclusion threshold 0.1 and AF2212 amino acid sequence as a query) revealed a statistically significant similarity with the AbrB superfamily of DNA-binding proteins (e.g. GI:15920210 with E-value ~ 0.071, 4^th ^iteration). The AbrB proteins have been identified as antitoxins in well-characterized TAS such as MazEF, Kis-Kid and PemIK [3,4]. The secondary structure of AF2212 and multiple alignment of this family support the PSI-BLAST results and reveal at least three β-strands that are most conserved in other AbrB superfamily members [51] (Figure 4C). The proteins of this family are found mostly in the genomes of thermophiles with only a few exceptions such as *Methanospirillum hungatei *(archaea isolated from bovine rumen) and *Lyngbya sp*. (a cyanobacterium).

**Figure 5:**
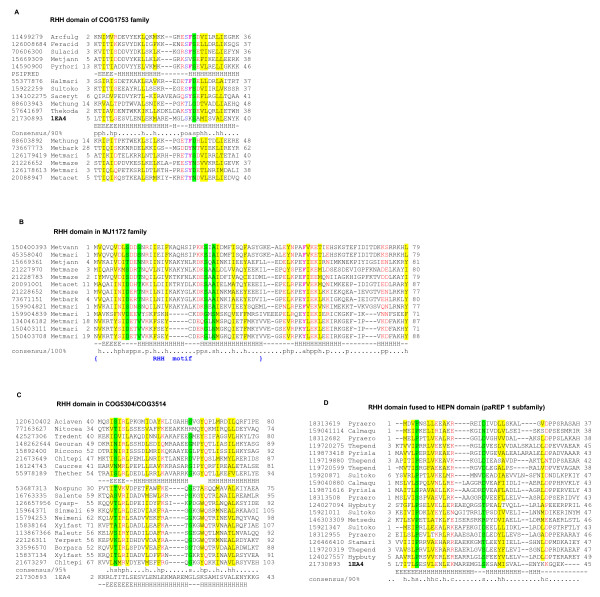
**Multiple alignments of distinct predicted antitoxin families containing ribbon-helix-helix (RHH) domains**. **A**. RHH domain of COG1753 family. **B**. RHH domain in MJ1172 family. **C**. RHH domain in COG5304/COG3514. **D**. RHH domain fused to HEPN domain (paREP 1 subfamily). Designations are as in Figure 2.

**COG1753 (DUF217) (20) **is an archaea-specific family of proteins that are often encoded within predicted operons with PIN family toxins. Notably, in several methanogens, this antitoxin is associated with RelE-like toxins rather than with PIN family toxins (e.g. MA0375- MA0376 system in *Methanosarcina acetivorans*). A PSI-BLAST search started with a query sequence of one from the above proteins (gi|126179419, with inclusion threshold E-value 0.01), detects RHH superfamily proteins after the second iteration (eg. gi|189499687 with E-value 3 × 10^-4^), and secondary structure prediction and multiple alignment support the presence of the RHH domain (Figure 5A).

**MerR (33) **is a well-characterized transcriptional regulator involved in stress response, especially, in heavy metal resistance [52]. We identified a distinct family of proteins that contain a HTH domain related to that of MerR and are associated with PIN-family toxins. These proteins belong to a distinct group of MerR-like DNA-binding domains known as truncated HTH domains that lack the Wing 2 region composed of two α-helices and the long dimerization helix [52,53]. In addition to the MerR-like domain, proteins of this family also contain a small domain at the N-terminus that might interact with the PIN domain (Additional file 3).

#### A new antitoxin family associated with RelE family toxins

One of the new families that we identified in association with RelE-like toxins is typified by the **MJ1172 **(COG7997) protein and so far found only in methanogens. The members of this family can be recovered using the sequence gi|159904821 as a query after 5^th ^iteration of PSI-BLAST with inclusion threshold 0.1. In this search, several proteins of the RHH superfamily appear slightly below the threshold (gi|186903258 with E-value 1.1). Secondary structure prediction for COG7997 proteins reveals a strand-helix-helix motif characteristic of the RHH superfamily (Figure 5B). The proteins of the RHH fold, such as RelB, are well-characterized antitoxins for RelE toxins [3,4]. Thus, it appears most likely that MJ1172-like proteins are a highly diverged family of RHH domain-containing antitoxins.

#### A new antitoxin family associated with MazF family toxins

We detected a specific family of small proteins associated with MazF toxin family that is predominantly present in proteobacteria and chlorobii/bacteroidetes groups and is typified by **XF1863 **(Figure 3). No functionally or structurally characterized homologs of these proteins were detected. Secondary structure prediction for this family does not precisely fit any characterized antitoxin families but indicates the presence of a strand-helix-helix motif, resembling the structure of RHH domains (Additional file 4). One of these proteins (gi|152585) is encoded on the Plasmid RSF1010 in a predicted operon with a small "unknown protein E" (gi|152584), and the two proteins have been reported to form a dimer that is involved in the regulation of the plasmid copy number [54]. These findings are compatible with the prediction that **MazF-XF1863 **proteins comprise a new TAS.

#### A new antitoxin family associated with Fic/Doc family toxins

A new family of predicted antitoxins associated with Fic/Doc toxins, typified by the *E. coli *protein **YhfG**, is present only in Enterobacteria. These small proteins are predicted to possess, mostly, α-helical structure and are enriched in positively charged amino acids, which is compatible with a DNA-binding capacity (Additional file 5).

### New toxins associated with known antitoxins

The antitoxin families are much less specialized than the known toxin families, that is, include numerous members that perform other functions in prokaryotic cells. In particular, many of these proteins are transcriptional regulators that are involved in the control of a variety of functional systems, mostly, related to stress response. This multifunctionality of antitoxins complicates prediction of new toxins solely on the basis of genomic association with antitoxin genes. Nevertheless, such cases are worth examining in search of putative TAS with novel molecular mechanisms that might merit experimental validation. Here we describe *in silico *evidence supporting the existence of several potential TAS with a novel toxin component.

#### The SMa091-like family of putative toxins associated with PHD antitoxins

PHD family antitoxins are found in association with three structurally unrelated toxin families, namely, Fic/Doc, PIN and RelE [13]. Our approach revealed a new α-proteobacteria-specific family of proteins encoded next to PHD antitotin genes; the family is typified by the SMa0917 gene that is located on the *Sinorhizobium meliloti *pSymA megaplasmid [55]. A PSI-BLAST search using this protein as a query with the inclusion threshold 0.1 detected proteins of the PemK/MazF family in the 6^th ^iteration (eg. gi|59801978 with E-value 0.016). The secondary structure prediction for the SMa0917-like proteins is compatible with the structure of PemK/MazF domains (Figure 2B). Thus, it appears that the SMa0917 family belongs to the PemK/MazF superfamily of mRNA interferases and represents the fourth group of toxins associated with PHD antitoxins.

#### A putative toxin family associated with RHH antitoxins

A predicted two-gene operon comprised of two families of small uncharacterized proteins (COG2929/DUF497 and COG5304/COG3514) is widespread in prokaryotes. We detected over 200 instances of this operon in a variety of bacteria representing most of the major bacterial lineages, and in several archaea. PSI-BLAST searches failed to identify reliable similarity of COG2929 proteins to any characterized proteins; however, the multiple alignment includes several conserved positions occupied by polar residues which is compatible with an enzymatic activity of the COG2929 family proteins (Figure 2C). The predicted secondary structure of these proteins is most similar to that of RegB/RelE nuclease superfamily, suggesting the possibility that COG2929 proteins are highly derived RelE-like interferases [56].

There are no functionally characterized proteins in COG5304 but several of these proteins are annotated as containing the RHH domain. Indeed, database searches identify a RHH domain in these proteins (PSI-BLAST search with gi|148262862 as a query with inclusion threshold 0.1 detects proteins of the RHH family after 5^th ^iteration, e.g. gi|93006664, with E-value 0.035; the RHH domain also can be detected in these proteins with any methods using comparison with profiles, like SMART or CDD). Secondary structure prediction revealed a C-terminal strand-helix-helix motif, in agreement with the PSI-BLAST results (Figure 5C).

Taken together, these observations, suggest that COG2929 proteins are toxin nucleases distantly related to Regb/RelE whereas COG5304 proteins are DNA-binding antitoxins containing the RHH domain. This operon is seen in several bacteriophages (*Burkholderia *phages phi52237 and phiE202) and plasmids (pLB, pNL14, pANL), suggesting horizontal mobility. Taken together, all these observations are compatible with the hypothesis that COG2929 and COG5304 proteins comprise a previously undetected TAS.

#### The DUF397 family of putative toxins associated with HTH domain-containing protein

The DUF397-HTH pair of genes encodes proteins that are most abundant in actinobacteria, with a specific expansion in *Salinispora tropica *CNB-440 (20 operon copies). This gene pair is present on several plasmids (plasmid pA387 from *Amycolatopsis benzoatilytica*, plasmid pNO33 of *Streptomyces albulus*; linear plasmid SCP1 of *Streptomyces coelicolor*, pNF1 of *Nocardia farcinica*). One of the genes in this pair (e.g. the SCO6130 gene from *S. coelicolor*) encodes a protein of approximately 300 amino acids that consists of an easily identifiable N-terminal HTH domain of the Xre family and an uncharacterized C-terminal domain.

The protein product of the second gene (DUF397, e.g. SCO6129) has been characterized previously as a potential pleiotropic regulator that affects morphogenesis, antibiotic production, and catabolite control in *Streptomyces *[57]. It has been reported that the BldB protein of the DUF397 family forms a dimer, is structurally flexible and regulates its own promoter [57,58]. Although it has been suggested that BldB contains an HTH motif [57,58], we were unable to validate this observation (Additional File 6). Considering all features of this gene pair, including non-uniform expansion of the two-gene operons in bacterial genomes, their presence on plasmids, the presence of the Xre domain in one of the encoded proteins and the pleiotropic regulatory effect, we suspect that the proteins containing the Xre domain function as antitoxins whereas DUF397 family proteins are novel toxins.

#### Zn-dependent proteases associated with HTH domains

Proteins of COG2856 (approximately 160 amino acids long) contain a conserved HEXXH motif that is the sequence signature of numerous families of metzincin Zn-dependent proteases [59] and show statistically significant sequence similarity to proteins of the Peptidase_MX (CL0150) family. This family of predicted Zn-dependent proteases is one of the most abundant gene families that form putative operons with HTH domain-containing proteins of the Xre family (eg. ydcM, COG2856 and ydcN, xre family HTH in *Bacillus subtilis *genome). In more than half of the cases, COG2856 domain and the Xre domain are fused within a single two-domain protein (e.g. *Mycobacterium tuberculosis *Rv2515c). Many of the fused protein genes are located in the same (predicted) operon with other toxins, mostly, of the PIN or RelE families (Table 2). These putative operons are abundant in various phages and prophages (*Staphylococcus *phage phiETA3, *Clostridium *phage phiC2, *Leptospira biflexa *temperate bacteriophage LE1, *Mycobacterium *phage Che9c, etc.) and several plasmids (*Sphingomonas *pCAR3, *Microscilla *sp. pSD15). Lineage-specific expansion of this pair of genes is seen in several bacteria, especially, Firmicutes (Figure 3) including the largest expansion in *Enterococcus faecalis *V583 (8 predicted operons, one fusion) and three copies of the operon encoded in several strains of *Streptococcus pyogenes*, bacteria with relatively small genomes.

These proteins have been previously implicated in the genetic control of bacterial suicide through the demonstration that induction of late genes of prophage PBSX causes cell death and the gene largely responsible for this effect is *xre *(which is encoded in one operon with ORF2 that belongs to COG2856) [60]. The mechanism of killing remains unclear but the connection of the expression of these genes with response to DNA-damaging agents has been established [61]. Furthermore, it is known that the Xre protein regulates not only its own expression but also the expression of other genes including a complex regulatory cascade downstream [60]. Considerable experimental data have been amassed on another protein of this family, the product of the *irrE*(*pprI*) gene (DR0167) of *Deinococcus radiodurans *which contains fused COG2856 and Xre domains. It was shown that IrrE/PprI protein is a key regulator of the *recA *gene induction after irradiation [62,63]. Unlike *recA*, which is strongly induced following irradiation [64,65], the *irrE *gene appears to be constitutively expressed, with no post-irradiation induction [64,66]. Also, the IrrE/PprI protein does not appear to bind the promoter region of *recA *or other induced genes [66]. These observations point to a pleiotropic effect of the expression of this gene pair, which is consistent with the data on the action of other TAS. The domain architecture of the COG2856-Xre fusion protein invokes the analogy with the well-characterized SOS response regulator LexA, an autoprotease and a repressor of a complex regulon that includes, among other genes, several TAS operons, [67-69]. In agreement with this hypothesis, it has been recently shown that the COG2856 protein ImmA (YdcM) from *B. subtilis *is required for the proteolytic inactivation of the Xre family protein ImmR, the repressor of the transposable element ICEBs1 [70]. Considering the strong link between the COG2856-Xre fusion protein and the RelE and PIN toxins, it appears most likely that this protein functions as a protease that cleaves a Xre family repressor which is either the antitoxin for the RelE/PIN toxin or a part of another, more complex regulatory cascade involving other stress-response genes. These findings suggest the existence of a novel, three-component TAS, with a more complex regulatory circuit than those of the currently described TAS. Experimental validation of this prediction and elucidation of the specific regulatory targets of the Xre repressor associated with the COG2856 family protease will be of considerable interest.

#### The RES domain associated with Xre HTH domains

The RES domain (~160 amino acids long) was named after three amino acids, arginine, glutamate and serine, which are conserved in the sequences of the respective proteins, suggesting a potential enzymatic activity . In most cases, genes encoding RES-domain proteins form predicted operons with genes of the uncharacterized COG5642. A PSI-BLAST search shows that the COG5642 proteins contain a Xre family HTH domain (this relationship can be easily demonstrated using any COG5642 query and running 2–3 search iterations).

The operons containing these genes are often present on plasmids including pSymB of *Sinorhizobium meliloti *1021, pKB1 megaplasmid of *Gordonia westfalica*, pCAR1 of *Pseudomonas resinovorans*, pTi-SAKURA of *Agrobacterium tumefaciens*, plasmids pRL11, pRL9 and pRL12 of *Rhizobium leguminosarum*. Lineage-specific expansions of this gene pair are seen in many genomes including *R. leguminosarum *(4); *S. meliloti *1021 (4); *Azotobacter vinelandii *(4); *Burkholderia vietnamiensis *G4 (5). Despite the lack of conclusive evidence on the activity of RES-domain proteins, it is tempting to speculate that this domain is an uncharacterized nuclease, and accordingly, COG5654-Xre (COG5642) gene pair encodes a novel TAS that could be a plausible target for further experimental analysis.

### Mobile two-gene modules with conflicting lines of evidence

There are several conserved two-gene operons identified by our approaches (Figure 1) that possess some but not all of the characteristic features of TAS or else possess experimentally characterized properties that do not seem to support the TAS prediction. Some of these modules could be TAS with novel mechanisms whereas others are likely to represent other classes of stress-response systems including determinants of antibiotic resistance.

#### The MNT-HEPN system

**Figure 6:**
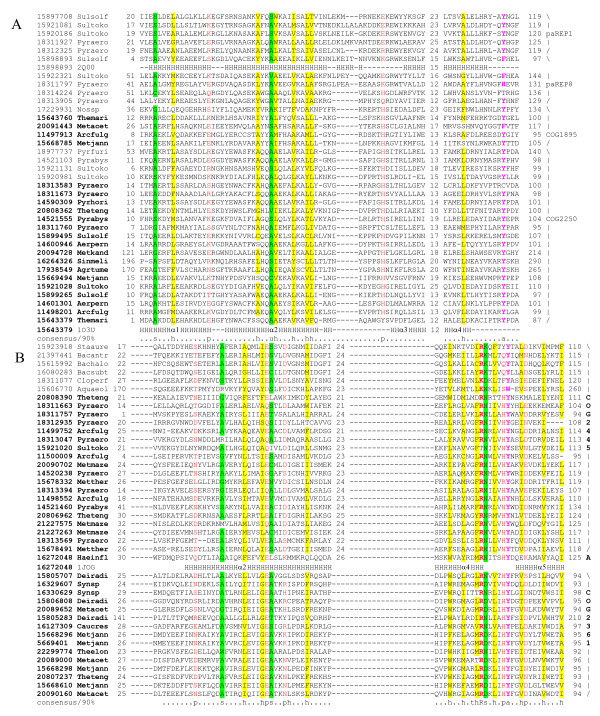
**Multiple alignment of the conserved cores of two distinct families of HEPN domains**. The distinct subfamilies of HEPN domains are indicated by brackets on the right. Designations are as in Figure 2.

The MNT-HEPN pair is by far the most widespread in this class of mobile two-gene modules. MNT and HEPN genes that are predicted to form an operon are among the most abundant genes in many archaeal genomes, but their functions remain enigmatic [36-38,46]. Several combinations of MNT and HEPN domain subfamilies were detected by the procedure for finding "mobile" COGs that form conserved two-gene operons (Table 1). Hence two arguments in support of the hypothesis that the MNT-HEPN pair is a hitherto unrecognized TAS: 1) these genes form a two-component system; 2) the predicted MNT-HEPN operons appear mobile given their non-uniform distribution across genomes. In addition, both MNT and HEPN are small proteins, typically, less than 150 amino acids in length. The two MNT subfamilies (COG1708 and COG1669) are well-conserved [37] whereas HEPN domain families are much more diverged, especially, COG2250 and COG2445/COG2361 groups (Figure 6). Structures of representatives of both HEPN subfamilies have been solved and shown to belong to the nucleotidyltransferase substrate-binding domain superfamily of the four-helical bundle fold [71]. Therefore, unlike most previously identified antitoxins, HEPN is, probably, not a DNA-binding domain. The situation is similar to that observed with the HicAB system where HicB domains themselves, most likely, are not DNA-binding, but some are fused either to RHH domains or to Xre family HTH domains. The present comparative-genomic analysis revealed a subfamily of HEPN-domain-containing proteins that contain a fusion of HEPN and RHH domains (Figures 6 and 5D). This particular subfamily of HEPN proteins is expanded in *Thermoproteales*, especially, in *Pyrobaculum *(paREP1 family, *Pyrobaculum aerophilum *repetitive family 1 [72]) although, surprisingly, none of these genes forms an operon with MNT. Nevertheless, the fusion with the RHH domain provides an indirect connection between HEPN and known antitoxins.

The molecular mechanism of action of the putative MNT-HEPN TAS remains unknown but it is tempting to speculate that the HEPN-MNT complex targets nucleic acids analogously to numerous other TAS. More specifically, the predicted toxin, MNT, might nucleotidylates RNA molecules to tag them for degradation whereas the predicted antitoxin, HEPN, could inhibit this reaction, perhaps, via non-productive nucleotide-binding. It seems possible that there is a DNA-binding component encoded in-trans that cooperates with MNT-HEPN, making it a three-component system.

Several HEPN-MNT units are located on plasmids, such as the Rms149_p38-39 pair of genes on *Pseudomonas aeruginosa *plasmid Rms149 or a HEPN-MNT fusion on *Agrobacterium tumefaciens *plasmid pAgK84. Furthermore, in some closely related species, it is possible to trace very recent insertions or deletions of the HEPN-MNT module. For instance, in *Deinococcus radiodurans*, a HEPN-MNT (DR0679-DR0680) gene pair is present within a gene cluster that mostly consists of GNAT-family acetyltransferase-related genes (DR0678-DR0684), whereas *D. geothermalis *lacks this putative operon in the otherwise syntenic orthologous locus (Dgeo_2060-Dgeo_2065). An analogous case is seen in two strains of Thermus themrophilus where a MNT-HEPN module is inserted within the lysine biosynthesis operon in the HB8 strain but not in the HB27 strain. This apparent recent mobility of the HEPN-MNT modules is compatible with the hypothesis that these genes comprise a novel TAS.

**Figure 7:**
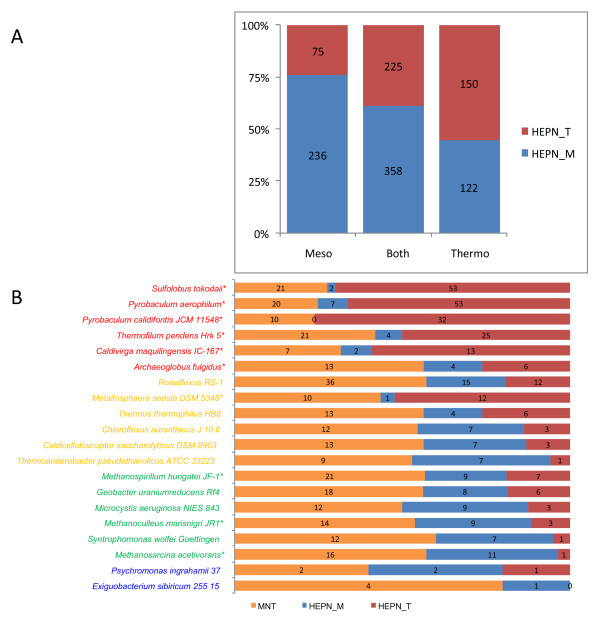
**Relative abundance of HEPN_T, HEPN_M and MNT domains in thermophiles and mesophiles**. **A**. The total number of HEPN-MNT pairs in hyperthermophiles and thermophiles ("Thermo"), mesophiles and psychrophiles ("Meso") and all ("Both") genomes. **B**. The number of HEPN_T, HEPN_M and MNT genes in selected genomes. Font color indicates the temperature preference: red – hyperthermophiles; gold – thermophiles; green – mesophiles; blue – psychrophiles. Asterisks indicate Archaea.

The predicted HEPN-MNT TAS shows an unusual phyletic distribution. We have shown previously that HEPN domains of a particular subfamily (COG2250) is represented, mostly, in thermophiles and can be considered one of the most prominent genomic correlates of the thermophilic life style [73]. The distribution of the two HEPN subfamilies across the available genomes of thermophilic and mesophilic prokaryotes (Figure 7A) is highly non-uniform (χ^2 ^p-value of 5 × 10^-17^). Considering the lineage-specific expansion in Thermoproteales of the "thermophilic" form (paREP subfamily, Figure 6) of the HEPN that it not associated with MNT, the possible correlation with thermophilic phenotype might be even stronger than previously noticed (Figure 7B). These intriguing observations suggest that there might be systematic differences between related TAS in thermophiles compared to mesophiles.

As shown above, the MNT-HEPN modules possess the major characteristics of TAS, most notably, the persistence of a two-gene module encoding small proteins, the strongly non-uniform distribution among genomes and high horizontal mobility. However, this module also shows some features that are not seen in experimentally characterized TAS: the putative toxin is not a nuclease (although it could be a nucleic-acid-modifying enzyme) whereas the putative antitoxin is unlikely to be a transcription regulator. Therefore the possibility remains that MNT-HEPN is not a bona fide TAS but rather belongs to a broader class of mobile stress response systems. For instance, MNT-HEPN potentially could function as an antibiotic inactivation system, via nucleotidylation of antibiotic molecules.

#### Other TAS-like gene pairs

Another mobile two-gene module consists of genes for a Xre-family HTH domain-containing protein and a protein of an uncharacterized family typified by *E. coli *YgiU. Two YgiU-like subfamilies (Additional File 7) are linked to two distinct families of HTH proteins. The proteins of the first subfamily, represented by YgiT, contain an additional N-terminal Zn finger domain, whereas the second subfamily (typified by *Lactococcus *phage bIL311 protein Orf21) is characterized by the fusion of an N-terminal HTH domain and a domain of the GepA (genetic element protein A) family. The latter family together with its YgiU-like counterpart is present mostly in phages and prophages of Firmicutes, whereas the *gepA *gene was detected next to a genomic region enriched in TA genes in *Dichelobacter nodosus *[74]. We detected no large expansions of this gene pair but several genomes contain two or more copies (two in *Thermoanaerobacter pseudethanolicus*, and three *Beggiatoa sp*. and *Geobacter uraniireducens*). The B3022(YgiY) protein is involved in the regulation of cell motility and biofilm formation in *E. coli *and accordingly was renamed motility quorum-sensing regulator, MqsR [75]. Furthermore, it was shown that MqsR overexpression has a toxic effect on *E. coli *growth, which is partially reduced by the YgiT/B3023 mutation, and that YgiT/B3023 is a regulator that induces expression of another GntR family gene which in turn controls production of colanic acid, a specific exopolysaccharide that is an important factor of biofilm formation [76]. However, many bacteria (and, of course, phages) that encode this pair of genes are not motile and do not form biofilms, so this module might also coordinate expression of genes with other functions, perhaps, via a TAS-like mechanism such as interference with mRNA translation.

Similar reasoning seems to apply to another pair of genes detected in our search for putative TAS, namely, the repressor and antirepressor that are present in the genomes of many bacteriophages, but not in bacterial genomes. The antirepressor protein contains two domains: an N-terminal Bro-N and a C-terminal Ant or KilA-C family domains [77]. Experimentally characterized antirepressor genes are continuously expressed, interact with the corresponding repressor genes, and together determine the state of the phage (that normally exists as a plasmid in the bacterial cell) by regulating the lytic genes expression [78]. It was also shown that some of the proteins containing Ant domains are toxic to bacteria [79]. These characteristics are reminiscent of TAS. However, given that the antirepressor proteins do not contain domains typical of toxins, it seems likely that this system functions analogously to the COG2856-Xre system, that is, that the formation of the Bro-Xre complex regulates the expression of a still unknown third component.

The only stable two-gene combination that includes HTH DNA-binding domains other than those of the Xre family is the combination of ArsR-like proteins with COG3832 (Table 2). This predicted operon shows a non-uniform distribution typical of TAS, with several prominent expansions (Table 1) including 10 in *Rhodococcus *RHA1, 9 in *Solibacter usitatus*, 7 in *Mesorhizobium loti *and *Janibacter sp*., and numerous genomes with two to six copies, but so far was not detected in plasmid or phage genomes. The ArsR family repressors are well-characterized regulators of cellular response to stress induced by heavy metals [80]. These repressors usually are associated with proteins responsible for detoxification, often forming two-gene operons ([80] and Table 2). Most of the characterized repressors of this family contain conserved metal-binding motifs [80] that, however, are missing in the ArsR subfamily associated with COG3832 (Additional File 8). Eukaryotic proteins of the COG3832 family play an important role in stress response through the activation of the ATPase activity of HSP90 [81], hence the name AHA1 (activator of Hsp90 ATPase). The N-terminal domain is responsible for binding to HSP90 [82] whereas the function of the C-terminal domain remains unknown. Another protein of this family, CalC, is involved in bacterial resistance to the DNA-damaging agent calicheamicin, via the disruption of the reactive bonds of the calicheamicin molecule accompanied by inactivation of CalC [83]. The structure of CalC has been solved, and it was shown to belong to the TBP (TATA-binding protein) fold [84] and, specifically, to the START superfamily [85] which unites various lipid-binding proteins, bacterial polyketide cyclases/aromatases and plant stress/pathogen response proteins, some of which possess RNAse activity [86]. Thus, although the role of this module in antibiotic resistance appears to be its most plausible function, the potential of this system as a novel TAS also deserves investigation.

Another unexpected observation is the strong link between both Xre family HTH and RHH domain-containing protein and N-acetyltransferases of the GCN5-related (GNAT) superfamily (Table 2). Enzymes of this superfamily catalyze the transfer of the acetyl group of acetyl coenzyme A to a variety of substrates, including diverse small molecules and proteins. In prokaryotes, GNAT acetyltransferases are involved in a variety of cellular functions including regulation of translation by acetylation of ribosomal proteins, arginine and UDP-N-acetylmuramyl pentapeptide biosynthesis, and antibiotic resistance (for review, see [87]). We identified several GNAT-RHH operons located on plasmids (*Azoarcus sp*. EbN1 plasmid; *Shigella flexneri *large virulence plasmid) and also observed many lineage-specific expansions of these operons 6 copies in *Photorhabdus luminescens*, and 4 in *Salmonella enterica *and *Rhodopseudomonas palustris*. None of the predicted GNATs associated with DNA-binding proteins has been experimentally characterized. The most similar experimentally studied GNATs are involved in resistance to tabtoxinine-β-lactam, the β-lactam phytotoxin, and antibiotic of *Pseudomonas syringae *([88] and Additional file 9). Thus, as in the case of COG3832-ArsR association, it seems most likely that GNAT-RHH and GNAT-Xre operons are involved in antibiotic resistance rather than being a bona fide TAS.

The similarity of the genomic characteristics of these modules to those of bona fide TAS suggests that mobile, regulated two-gene modules could be broadly involved in diverse forms of stress response in prokaryotes.

### The distribution of TAS-like systems in prokaryotic genomes

Previous surveys of the occurrence of TAS in prokaryotes revealed that they are typically absent in organisms with small genomes most of which are parasites or symbionts [26,27]. The addition of the new predicted TAS identified in this study has not changed this conclusion (Additional File 10). In particular, we still do not detect any TAS in *Borrelia*, endosymbionts of the gamma- and alpha- proteobacterial lineages, and the majority of Mollicutes (mycoplasmas). Among archaea, no TAS were identified in Thermoplasmatales, several methanotrophs with small genomes, and the only known symbiotic archaeon, *Nanoarchaeum equitans*.

**Figure 8:**
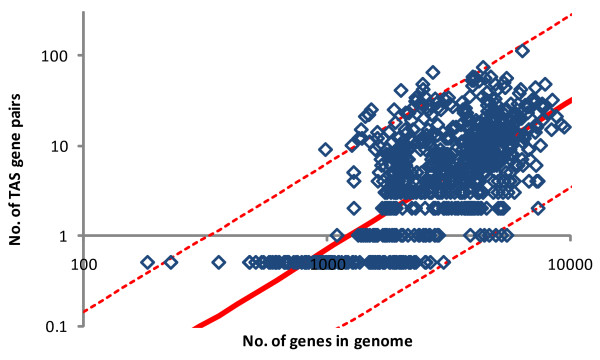
**The relationship between the number of detected TA pairs and genome size**.

It has been proposed that the absence of TAS in prokaryotes with small genomes could be due to their relatively simple life style in stable environmental conditions [26]. An alternative explanation, however, could be that the observed distribution is a simple consequence of the general "laws" of scaling of differential functional categories of genes with genome size [89-92]. We plotted the number of detected TA gene pairs against the genome size of prokaryotes (Figure 8) and detected a strong positive correlation (Spearman rank correlation 0.61, *p *<< 10^-10^). A maximum likelihood estimate (see Additional file 11) indicates the scaling exponent value of 1.64. This value is higher than the exponents of most of the other functional classes but significantly lower than the (near) quadratic scaling that is characteristic for transcriptional regulators and components of signal transduction systems [90,91]. Given the high variance of the abundance of TAS genes, the total absence of TAS in some of the genomes with up to ~3100 genes is expected within the 95% confidence interval (Figure 8). Given that the largest genome with no TAS detected, *Prochlorococcus marinus *MIT 9303, contains 2997 genes, it is possible that the absence of TAS in certain prokaryotes is a simple consequence of the allometric scaling with genome size and does not require special biological explanations.

The other significant factors (see Additional File 10) that appear to affect the distribution of TAS among genomes independently of the genome size (Table 4) are the host taxonomy (Archaea generally possess more TAS than Bacteria relative to the genome size, *t*-test p-value of 1 × 10^-4^), optimal growth temperature (thermophiles tend to be enriched with TA genes compared to mesophiles and psychrophiles, *t*-test p-value of 5 × 10^-4^) and environment (terrestrial and multi-environmental microorganisms typically possess significantly fewer TAS than predicted from the genome size whereas prokaryotes living in aquatic, host-associated and specialized environments often contain a greater number of TAS than predicted, *t*-test p-value of 2 × 10^-3^). The three factors seem to be statistically independent (that is, each retains its significance after adjusting for the others, see Table 4), although the independence between the taxonomic affiliation and temperature preference is difficult to prove conclusively because of the abundance of thermophiles among Archaea.

**Table 4:** Association of TAS with ecological features of prokaryotes

Group 1/Group 2	Group 1 median	Group 2 median	T-test *p*-value
*Residuals after scaling by genome size*			

Archaea/Bacteria	0.39	0.00	**0.0001**
(hyper)thermophiles/meso- & psychrophiles	0.34	0.05	**0.0005**
Terrestrial & multi-environmental/other	-0.01	0.05	**0.0022**

*Residuals after scaling by genome size and adjustment by taxonomy*			

(hyper)thermophiles/meso- & psychrophiles	0.16	-0.01	0.0592
Terrestrial & multi-environmental/other	-0.05	0.00	**0.0157**

*Residuals after scaling by genome size and adjustment by temperature*			

Archaea/Bacteria	0.22	-0.01	**0.0133**
Terrestrial & multi-environmental/other	-0.05	0.00	**0.0180**

*Residuals after scaling by genome size and adjustment by environment*			

Archaea/Bacteria	0.30	-0.01	**0.0003**
(hyper)thermophiles/meso- & psychrophiles	0.25	-0.01	**0.0016**

Bold type highlights *p*-values significant at the 0.05 level.

With respect to the representation in major bacterial and archaeal lineages, the TAS (Additional file 12) ranged from nearly ubiquitous (e.g. HEPN-MNT, AbrB-PIN, PIN-RHH) to clade-specific (COG2886-RelE in Cyanobacteria, DUF397-Xre in Actinobacteria, MazF-PHD in Alphaproteobacteria and Fic-YhfG in Gammaproteobacteria). The distribution of TAS across phyla is distinctly non-uniform, with many systems significantly over- and underrepresented in various taxa (Figure 3). We identified no archaea-specific TAS but many bacteria-specific ones; this observation seemed somewhat unexpected considering the greater abundance of TAS in archaea although it could simply reflect the greater diversity of available bacterial genomes. The repertoires of TAS (and TAS-like systems) in the hyperthermophilic bacterial phyla, Aquificae and Thermotogacae, resembles that in the Archaea, primarily due to the high abundance of the "thermophilic" version of the HEPN-MNT system and the near-absence of typical bacterial systems. Among bacteria, Bacteroidetes-Chlorobi, Alpha- and Gammaproteobacteria and Cyanobacteria possess the greatest variety of TAS (10–13 statistically overrepresented gene pairs). Among taxa with numerous sequenced genomes, Firmicutes (especially Bacilli) are characterized by a particularly low TAS diversity (3 statistically overrepresented pairs). The most uniformly distributed systems are AbrB-RelE, GNAT-Xre and AbrB-MazF. On the opposite end of the spectrum, several widespread systems, such as HEPN-MNT, PIN-RHH, COG2886-PIN and PHD-PIN, have sharply contrasting distributions (i.e. either significantly over-represented or underrepresented in different host taxa).

### Co-occurrence of toxins and antitoxins in TAS operons

**Figure 9:**
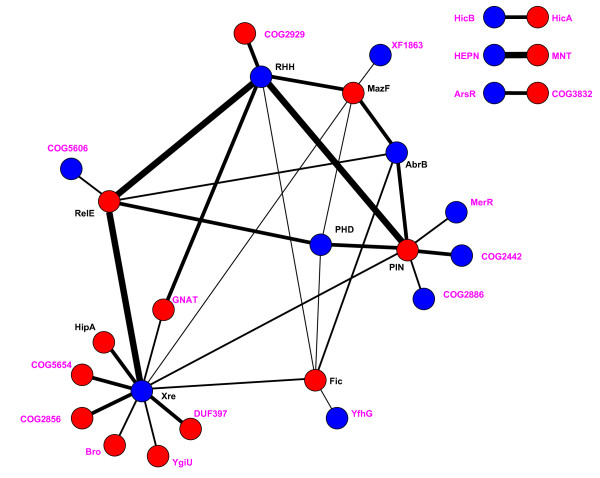
**Graph of relationships between different families of toxins and antitoxins**. Known (black) and predicted (magenta) toxins (red circles) and antitoxins (blue circles) and their operon organizations. Lines connect genes with 5 or more two-component operons found; thickness of a line is proportional to the frequency of the respective operon.

The relationships between toxins and antitoxins can be represented by a graph with edges connecting genes that form TA pairs (Figure 9). In a striking demonstration of the versatility and modularity of the TAS, most of the known and predicted TA genes belong to a connected network that covers 87% of the detected TAS. Three (putative) TAS (HEPN-MNT, HicA-HicB and ArsR-COG3832) occur strictly as unique pairs and are never involved in other TA combinations (except for several protein domain fusions discussed above). The principal hubs of the TAS network are two toxins (PIN and RelE) and two antitoxins (Xre and RHH) that, taken together, participate in 76% of all TAS. Accordingly, these four superfamilies have the greatest diversity of antitoxin (toxin) partners (7, 6, 13 and 6, respectively). The three most common TAS (RelE-Xre, PIN-RHH and RHH-RelE; 29% of the total number of systems) are composed entirely of these four genes.

The versatility and the modular character of the TAS notwithstanding, the combination of toxins and antitoxins is highly selective, even when only the connected component of the network is considered. Compared to random expectations based on overall abundance of genes, some TA pairs are either underrepresented (e.g. PIN-Xre or AbrB-RelE) or conspicuously absent (for instance, pairs involving exclusive toxin partners of Xre or exclusive antitoxin partners of PIN) whereas other pairs are overrepresented (AbrB-Fic is over three times more frequent than expected).

### Variability of TAS in closely related genomes

We examined the distribution of TAS in the 41 sets of closely related prokaryotic genomes (Alignable Tight Genomic Clusters, ATGC [93,94]). In 33 of the ATGC at least one of the 37 TAS was detected in at least one of the members. For all identified TAS, the standard deviation of the number of occurrences was computed within each ATGC and the average of this value was calculated across all TA pairs in this ATGC (excluding pairs completely absent from this ATGC) to obtain a single, ATGC-specific measure of variability. As a control, all proteins of the 163 ATGC members were assigned to COGs [33], a random sample of 37 COGs with the mean protein length less or equal to 150 amino acids was selected, and the variability of "regular" COG members was estimated for these 33 ATGCs in the same manner (see Additional file 11). In 32 of the 33 ATGCs, the variability of TAS significantly exceeded that of other COGs (the equal variability hypothesis is rejected with *p*-value of 4 × 10^-9^). Thus, the genomic occurrence of TAS shows exceptional variability even at close evolutionary ranges.

### Distribution of TAS operons on bacterial and archaeal chromosomes

**Figure 10:**
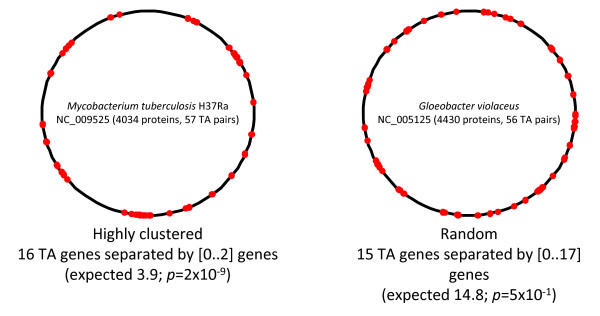
**TAS in selected genomes**. Red dots show the approximate position of TAS genes on the circular chromosomes.

Random, independent positioning of TAS pairs on prokaryotic chromosomes would produce an approximately exponential distribution of inter-TAS distances. Both the individual TA systems and aggregated data do not statistically differ from the random expectation (χ^2 ^test of observed vs. expected distributions of inter-TAS distances was performed for distance thresholds approximately corresponding to 25-th, 33-th and 50-th percentiles of the respective observed distributions). However, in many genomes a statistically significant excess of closely spaced TAS pairs (TAS "islands") was detected (Table 5). As in the majority of these cases the closely-spaced TA gene pairs belong to different classes of TAS systems, tandem duplication cannot explain the observed pattern. Possible explanations that are not mutually exclusive include preferential incorporation of TAS in a particular chromosome region and/or HGT of TAS "cassettes" consisting of multiple TA pairs. Conceivably, many prokaryotic genomes contain stress-response islands comparable to pathogenicity of symbiogenesis islands. Examples of essentially random and highly clustered TAS distribution are shown in Figure 10.

**Table 5:** TAS "islands" in prokaryotic genomes.

ACC no.	Genome	TAS pair	NO. of pairs	distance threshold	No. observed	No. expected	Chi2
NC_005070	Synechococcus sp. WH 8102	all	11	3	6	0.3	82.79
NC_011138	Alteromonas macleodii 'Deep ecotype'	all	19	3	6	0.5	48.27
NC_010842	Leptospira biflexa serovar Patoc strain 'Patoc 1 (Ames)'	all	22	3	7	0.7	47.09
NC_009565	Mycobacterium tuberculosis F11	all	46	2	12	2.1	43.43
NC_004757	Nitrosomonas europaea ATCC 19718	all	48	3	18	4.5	40.8
NC_009525	Mycobacterium tuberculosis H37Ra	all	57	3	16	3.9	36.41
NC_002755	Mycobacterium tuberculosis CDC1551	all	52	3	14	3.2	35.89
NC_008639	Chlorobium phaeobacteroides DSM 266	all	28	2	8	1.2	35.71
NC_008740	Marinobacter aquaeolei VT8	all	15	7	5	0.5	31.72
NC_002945	Mycobacterium bovis AF2122/97	all	53	3	14	3.5	30.44
NC_007484	Nitrosococcus oceani ATCC 19707	all	32	3	9	1.7	28.88
NC_008769	Mycobacterium bovis BCG str. Pasteur 1173P2	all	58	3	15	4.2	27.66
NC_000962	Mycobacterium tuberculosis H37Rv	all	53	4	14	4.1	23.22
NC_010803	Chlorobium limicola DSM 245	all	26	5	10	2.4	23.02
NC_010602	Leptospira biflexa serovar Patoc strain 'Patoc 1 (Paris)'	all	20	8	7	1.4	20.53
NC_010831	Chlorobium phaeobacteroides BS1	all	42	3	12	3.5	20.14
NC_007677	Salinibacter ruber DSM 13855	all	13	8	5	0.8	19.39
NC_008212	Haloquadratum walsbyi DSM 16790	all	10	5	3	0.3	19.31
NC_000917	Archaeoglobus fulgidus DSM 4304	all	30	3	8	1.8	18.79
NC_010161	Bartonella tribocorum CIP 105476	all	22	14	12	3.9	18.33
NC_011060	Pelodictyon phaeoclathratiforme BU-1	all	65	5	23	10.3	17.24
NC_008698	Thermofilum pendens Hrk 5	HEPN-MNT	14	3	4	0.7	10.92
NC_000917	Archaeoglobus fulgidus DSM 4304	AbrB-PIN	12	3	4	1.3	4.18

## Discussion

**Figure 11:**
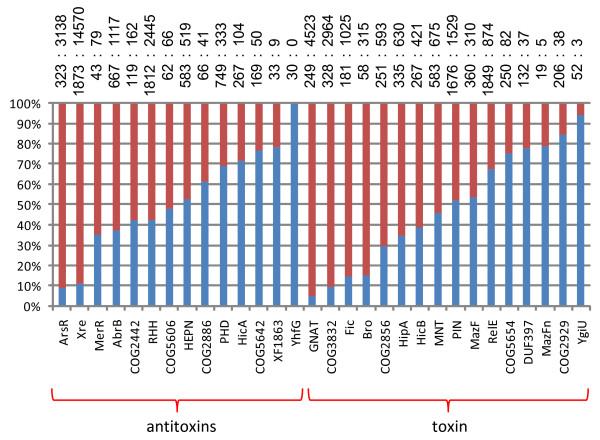
**Fractions of solo and two-gene operon occurrences for each family of toxins and antitoxins**. Red, fraction of solo genes; blue, fraction of genes in (predicted) operons.

"Classic" TAS were defined as two-component systems with a stable toxin and an unstable antitoxin encoded in the same operon and acting as an "addiction" mechanism, that is, requiring constant (over)production of antitoxin for microbial cell survival. The findings reported here reinforce the power of these organizational and functional principles through the discovery of numerous potential new TAS. However, the accumulating data also increasingly indicate that many TAS-like systems do not fit this paradigm. In particular, toxin expression could be extrinsically regulated ([3,6,68]) when the antitoxin is encoded *in trans*. First experimental evidence of this possibility was reported recently when the inhibitory effect of a chromosomally encoded antitoxin on a plasmid-encoded toxin was demonstrated [25]. This type of regulation might explain the unexpected high abundance of solo toxins and antitoxins – over 50% of the genes in the largest families – a finding that cannot be explained solely by mis-annotation of small ORFs (Figure 11). An alternative or, more realistically, additional explanation of this finding is that solo homologs of toxins and antitoxins perform functions distinct from those of TAS such as transcriptional regulation of diverse operons by antitoxin homologs. In addition, the antitoxin function can be performed by a small RNA as in Type I TAS ([68]).

In principle, some TAS might function as one-component or three-component systems. One example of a likely solo toxin is the cyanobacteria-specific Uma2 family (COG4636), that was previously described in connection with TAS [13] and predicted to be an endonuclease of the PD-(D/E)XK superfamily [95,96]. The members of this family are highly abundant in cyanobacteria but are only rarely associated with known antitoxins and instead mostly form cassettes of paralogous genes (Table 2). "Normal" gene regulation systems can also use the combination of stable and unstable components (as probably is the case of Xre-Bro system [78]) whereas TAS with more stable antitoxins might also function as generic regulators (as might be the case for the Xre-COG2856 and/or ArsR-COG3832 associations).

Conceivably, as demonstrated by the case of acetyltransferases associated with HTH and RHH domains, the class of mobile operons resembling TAS could be quite broad, including in particular antibiotic resistance systems. The MNT-HEPN, arguably, the most remarkable two-component system identified here, considering its dramatic overrepresentations in thermophilic archaea, is a case in point: the currently available data hardly allow one to determine whether this is a bona fide TAS or an antibiotic resistance system. The MNT-HEPN system emerges as the prime target for experimental study that will distinguish between the two possibilities. It should be noted that, although potentially mechanistically distinct, the TAS activity and antibiotic resistance can be biologically linked considering that many TAS confer resistance to antibiotics to bacterial cells, primarily, by driving them into persistence [97,98].

On a more general note, the TAS obviously belong to the prokaryotic mobilome [89,99,100] as they are extensively, if not preferentially, spread via plasmid-mediated HGT. Like many if not most of the mobilome members, the TAS are not simply mobile but appear to behave like selfish elements: although they do not carry genes or signals required for autonomous replication, their entire life style is best conceptualized as a strategy ensuring their own maintenance and propagation. There are strong analogies between the TAS and other components of the mobilome, in particular, the restriction-modification systems (RMS) [101]. The RMS are well known to protect prokaryotic cells from heterologous DNA through the destruction of unmodified DNA molecules by the restriction component whereas the host DNA is modified by the modification component of the RMS. This principle of action is strikingly similar to that of bona fide TAS with the exception that, in the case of the RMS, damage to the host cell is prevented not by inactivation of the "toxin" but rather by protection of the target (DNA) via a specific modification. However, this mechanistic distinction should not overshadow the deep biological commonality between the TAS and the RMS that is manifested in the shared "poison-antidote" principle, and in the apparent selfishness of both classes of systems that involves extensive horizontal mobility and addiction properties. Indeed, although the RMS are not often considered as a type of TAS, this seems to be due more to tradition than to a true, fundamental difference between the two classes of systems [3].

The biological common denominator between TAS, RMS, phage abortive infection systems [102] and some other molecular systems, such as those involved in antibiotic inactivation, is that they contribute to various forms of stress response. The connection is reinforced by the recent demonstration that one of the phage abortive infection systems, ToxIN, functions as a protein-RNA TA pair [103]. These systems comprise a distinct domain of the mobilome that can be denoted the resistome (this term is currently used to denote the set of microbial genes that confer antibiotic resistance [104] but it seems to make sense to apply it more inclusively). For such systems, the boundary between selfishness and "normal" cellular function seems to be fuzzy and more a matter of convention than a real distinction. Indeed, TAS-like systems tend to make any replicon in which they reside addicted to themselves. In the case of plasmids, this is achieved by dramatically increasing the chances of transmission of TAS-carrying molecules during cell division. In the case of chromosomal TAS, the basis for addiction could be resistance to the plasmid versions of the same TAS that allows the TAS carrying cells to exclude the respective plasmids (antiaddiction [25]). In this case, it might be beneficial for the cell to stay addicted to the domesticated, relatively harmless chromosomal TAS rather than tolerate the TAS-carrying plasmids. A similar logic applies more generally: TAS-like systems promulgate their own survival by making cells that carry them resistant to antibiotics or other forms of stress. Furthermore, the addictive character of these systems increases the probability of their fixation after HGT, hence the extensive horizontal mobility that is simultaneously a telltale sign and the dissemination mechanism of all selfish genetic elements. Integration of TAS into "normal" cellular regulatory circuits also can be viewed as continuation of their selfish strategy [5]; however, as they are integrated deeper, the horizontal mobility tends to be restricted and traded for more stable vertical inheritance, gradually pushing these selfish elements towards the status of "regular" chromosomal genes. This "selfish altruism" or "responsible selfishness" of TAS-like systems seems to be paradigmatic of the mobilome and a key feature of the prokaryotic biome in general because in the prokaryotic world the mobilome and the "stable" chromosomes form a dynamic continuum [89].

## Conclusion

We report here the most detailed and comprehensive comparative-genomic analysis of type II TAS so far available (to our knowledge) by using two computational approaches that were specifically developed for TAS prediction and analysis on the basis of the signature features of TAS that typically comprise two-gene operons encoding two relatively small proteins one of which contains a DNA-binding domain. This analysis resulted in the prediction of 12 novel families of toxins and 13 novel families of antitoxins including families that are specific for distinct groups of archaea or bacteria, in particular, thermophiles. In addition, we discovered numerous solo genes for both toxins and antitoxins, a finding that suggest novel principles of TAS functioning, such as *in trans *regulation, or recruitment of toxins and antitoxins for other functions, or most likely, both of these phenomena. Some of the newly predicted two-operon modules might not function as bona fide TAS but rather as other types of stress-response systems that could be, for instance, involved in antibiotic inactivation. The prime case in point is the MNT-HEPN module which is among the most abundant genes in hyperthermophilic archaea. Experimental study of this and other novel TAS-like systems is expected to reveal the exact functions and to shed new light on the life style of these widespread prokaryotic genetic elements. The TAS-like systems are prominent and typical components of the prokaryotic mobilome, and the interplay between their selfish behavior, addictiveness, and integration into "regular" regulatory circuits of archaea and bacteria seems to be the epitome of the dynamic equilibrium between mobile and more stable parts of the prokaryotic pangenome.

## Methods

### Identification (prediction) of TAS: approach 1

For each COG from the in-house COG database containing 110 bacterial and archaeal genomes (available from the authors by request) the coefficient of variation (CV) for the number of the paralogs (to the exclusion of species with no genes in the given COG) was estimated in order to find the COGs with unevenly distributed "mobile" genes. The top 2000 COGs with the highest CV values (between 2.98 and 0.47) were mapped to the corresponding chromosomes and further checked if they are adjacent to the same COG at least 3 times in at least one genome. This resulted in further reduction of the number of candidates for further analysis to 315 COGs. All these COGs were analyzed one by one using the STRING program [34] to discard those that are parts of larger (>2 genes) (predicted) operons and those where one or both members of a pair were often associated with genes from other families.

### TAS identification: approach 2

Exhaustive PSI-BLAST [39] searches were performed for each protein family of known toxins and antitoxins, using a variety sequences from each family as queries (the iterative searches performed for protein larger that 100 amino acids with inclusion threshold 0.01 or for protein smaller that 100 amino acids with inclusion threshold 0.1 until the convergence or until the last iteration before the first known false positives appear). All genes identified by this procedure were mapped to the corresponding genomes; closely located (intergenic regions shorter than 100 bp) co-directed neighbors were collected. Neighbors were then clustered using BLASTCLUST (-L 0.5 -S 1.75) and further classified using CD-Search [40]; results obtained by overlapping CDD profiles were combined. Clusters larger than 20 were further checked on the case-by-case basis to determine whether they form a stable two-gene operon or a larger conserved cluster, and those that belong to larger predicted operons were discarded.

### TAS in completely sequenced genomes

All identified TAS genes were grouped by family and clustered using BLASTCLUST (-L 0.75 -S 1.0). Representatives of each cluster were used as queries in a BLAST search (e-value threshold of 0.01) against 750 completely sequenced bacterial and archaeal genomes available on the NCBI Microbial Genomes website at the time of this analysis (September 2008). Significant hits among proteins encoded in these genomes were classified as toxins or antitoxins; in case of multiple matches to different TA families, the protein was assigned according to the highest-scoring match to a TA query. Co-directed genes with adjacent chromosome locations belonging to different toxin/antitoxin families were recorded as a TA pair.

### Protein sequence analysis

Multiple alignments of all toxin and antitoxin were constructed using the Muscle program [105] followed by manual correction on the basis of the predicted secondary structure (PSIPRED program [106]) and PSI-BLAST-based local alignments. For the species abbreviations used in all the alignments, see Additional File 13.

### Estimation of the scaling parameters

Normally, the allometric scaling coefficient for two variables is easy to estimate as the slope of the straight line on the log-log plot of these variables. Even when both variables are natural numbers (e.g. number of genes belonging to a particular category vs. the genome size expressed as a total number of genes), this direct approach is applicable if the numbers themselves are sufficiently large to minimize the discretization effect [91]. In the specific case of TAS genes, however, this approach cannot be applied because of the overall low abundance of TAS pairs (111 of the analyzed genomes have only 1 or 2 TAS pairs and 119 have none). Obviously, zero values cannot be plotted on a log-scale or used to compute the coefficients of the regression curve; however, omitting the zero points would mean the loss of over one-sixth of the data. To escape this conundrum, we designed a model where the expected number of TAS genes is allometrically scaled with the number of genes in a genome and the observed numbers reflect discretization of log-normally distributed deviation from the expectation. Parameters of this model were obtained by maximization of the likelihood of the observed data (see Additional file 11).

### Tests for effects of taxonomic affiliation, temperature and environment

The expected number of TAS pairs for each genome was estimated using the allometric scaling formula with the parameters estimated as described above (*t' *= 1.64log(*l*)-5.08, where *l *is the total number of genes in the genome (see Additional file 11). At the first round of comparison, log-scale residuals (*r *= *t*-*t'*, where *t *is the logarithm of the observed number of TAS pairs) were compared between different groups of genomes using the two-tailed *t*-test with unequal variances. For genomes with no TAS pairs, the detected *t *was assigned the value of log(0.5); however, positive residuals were reduced to 0 (i.e. a genome with zero observed TAS pairs can have less, but cannot have more TAS than expected). The following partitions of genomes were explored: by taxonomy (Archaea and Bacteria); by temperature preference (hyperthermophiles, thermophiles, mesophiles and psychrophiles); by environment (aquatic, terrestrial, host-associated, specialized and multi-environmental). At the second round of comparison, group averages of the first-round residuals were further subtracted from the first-round residuals (that is, the mean of the residuals across Archaea was subtracted from all residuals for archaeal genomes); the second-round residuals were compared between groups using the two-tailed T-test with unequal variances.

## Competing interests

The authors declare that they have no competing interests.

## Authors' contributions

KSM and EVK initiated the study; KSM performed data analysis; YIW wrote the custom scripts and performed statistical analysis; KSM and YIW wrote the initial draft of the manuscript; EVK wrote the final version of the manuscript that was read and approved by all the authors.

## Reviewers' reports

### Reviewer 1

**Kenn Gerdes**, Newcastle University Medical School (Nominated by Arcady Mushegian).

General comments: Makarova et al. suggest two straightforward ways to identify potential novel TAS in prokaryotic genomes and identify potential new TAS in 750 completely sequenced genomes and the work appears as a valuable bioinformatics study of TAS and TAS related genes. The manuscript represents a huge amount of work that may serve as one basis for the more detailed and meticulous characterization of TAS. It may also serve as a starting point for the experimental tests of the proposed biological functions and properties of the new TAS.

In general, the manuscript is difficult to follow for non-specialists. Moreover, it is difficult to reproduce important findings. For example, in the huge (and very useful Table in AF10, how was the number of TAS pairs reached? And how was the number All Toxins reached in AF10? To solve the problem with the Table AF10, the Authors could provide one or two examples describing in detail how they reached these numbers. Alternatively, they could simply provide the identifiers of the genes that they suggest are TAS (GIs or similar) although this would represent some work. In this connection we were surprised to see that the three sequenced *E. coli *K-12 strains W3110, MG1655 and DH10B have 9, 15 and 16 predicted TA loci, respectively.

**Authors' response**: *This is a regular (and, yes, very detailed) research paper and as such most of it is not addressed to non-specialists (that is, non-microbiologists). We do not believe, however, that the paper qualifies as being "esoteric". This being said, we agree that better documentation is desirable, so we prepared Additional File *14*which contains the information on all individual toxin and antitoxin families, and toxin-antitoxin pairs*.

Most articles use TA loci, not TAS, to abbreviate toxin – antitoxin genes/systems. We think the former appears less esoteric and already accepted by different journals.

**Authors' response**: *We preferred to keep the succinct, three-letter acronym, rather than referring to loci each time. On the two occasions when the acronym is introduced, in the Abstract and in the Background section of the main manuscript, we added "(TAS, also referred to as TA loci)"*.

Finally, we find the manuscript in several places too speculative (see below) and that it appears hastily written.

Specific points:

1. Abstract: ....present evidence that HEPN/MNT "is likely to be TAS" is a serious overstatement – please rephrase to "raises the possibility" or something like that.

**Authors' response**: *We do not really agree that this was a "serious" overstatement but the language was changed to: "we present indications that the two-gene module that encodes a minimal nucleotidyl transferase and the accompanying HEPN protein, and is extremely abundant in many archaea and bacteria, especially, thermophiles might comprise a novel TAS."*

2. It is not clear to us why TA loci should be included in the "bacterial resistome". The experimental evidence that TAS function in persister cell formation is weak.

**Authors' response**: *The resistome here is broadly defined as the compendium of genes involved in various forms of stress response, so we think that in this context the inclusion of TAS is appropriate. Further, our own perusal of the work on the involvement of TAS in bacterial persistence, in some of which we were involved directly, does not suggest that the evidence is weak, even as we are prepared to defer to Dr. Gerdes on this account*.

3. To our view, it is a general misunderstanding that *mazEF *functions in PCD as claimed by one group working with *E. coli *as the model system. We and others have never been able to reproduce PCD by mazEF in *E. coli*. This confusion has been inflated by the recent finding that *mazF *of *Myxococcus xanthus *functions in PCD during the formation of myxospores. In Myxo, *mazF *is not part of a bona fide TA locus. Rather mazF-Mx interacts with the transcription factor MrpC encoded elsewhere on the chromosome suggesting that MazF-Mx was recruited to become a component of the developmental pathway that leads to Myxo spore formation. Thus, MazF-mediated PCD in Myxo is probably not a typical TA locus phenomenon.

**Authors' response**: *The reference to the PCD by mazEF in E. coli is quite cautious. With regard to Myxococcus xanthus, the original text was indeed less than precise. We modified it to indicate that it was a solo mazF that mediated PCD in the Myxococcus Xanthus development*.

4. The description of Selection criterium #1 was partly unclear to us because the CVs in Table 1 are low (between 1.1 and 0.5) whereas we would expect a high CV for TA loci. Please comment and explain better on this point.

**Authors' response**: *We added some details in Methods section and in Figure *1*in order to clarify this. Specifically, we first selected 2000 of ~15000 COGs (with CV range from 2.98 to 0.47) and ran an automatic procedure to select only those that have a relatively conserved neighbor regardless of the CV. This step returned 315 pairs of COGs, and all these pairs were examined one by one. The CV values for all these 315 gene pairs are given in Additional File *1. *The known TAS, as can be seen in Table *1*and Additional File *1, *do not have extremely high CV values but many of them are within the analyzed CV range. Both comparative-genomic approaches employed here aimed at the detection of new "major" TAS, but not at the comprehensive identification of all representative of known TAS. The latter task was mostly performed by the PSI-BLAST analysis of the protein sequences encoded in 750 complete prokaryotic genomes (Additional File *10*and new Additional File *14) *once we have delineated all families of interest, with the caveat that we did not attempt to search for missed ORFs*.

5. Table 2: It is not entirely correct to call PIN domain proteins for "RNA interferases" – the evidence in the literature is derived from in vitro experiments only, and we have not found nuclease activity with two enteric VapCs, neither in vivo nor in vitro (Winther & Gerdes, in press).

**Authors' response**: *PIN domain proteins are not described as "RNA interferases" but rather as nucleases. In order to be even more cautious, in the revision, PINs are denoted "(predicted)" nucleases" at first mention*.

6. We identified two TA loci in *N. equitans *(VapBC loci; [35]) and so far we have not yet identified any archaeal genome without at least one TA locus.

**Authors' response**: *We also identified two PINs (VapC) in N. equitans (see Additional File *14) *but not the corresponding antitoxins that apparently have not been annotated in this genome as indeed follows from the Supplementary Material in *[35]. *As mentioned above, no attempt was made to annotate missing ORFs. That apart, we did not detect any TAS in any of the available Thermoplasma genomes, so there seem to be archaea devoid of TAS*.

7. We strongly favor the idea that the lack of TA loci in almost all obligate intracellular organisms have a biological background and is not just a "statistical coincidence". Most strikingly, *Mycobacterium tuberculosis *has more than 60 TA loci, whereas *M. leprae *has none! Since the genome of *M. leprae *was derived from that of *Mtb *by massive genome reduction, this must mean that the selection pressure to retain TA loci in *M. leprae *was lost. In turn, this observation correlates with the obligate intracellular life-style of contrasted by that of *Mtb *that exists both intra- and extracellularly.

**Authors' response**: *We did not claim that the lack of TAS in intracellular parasites and symbionts is a "statistical coincidence". Indeed, the genomes of these bacteria are usually highly reduced and thus are expected to contain fewer TA loci. We show that given the scaling of the number of TAS with genome size, the hypothesis that TAS are missing from genomes of the currently known intracellular organisms for purely stochastic reasons cannot be statistically rejected. Hence there is no evidence of the existence of a selective pressure to lose TAS in these organisms. Such pressure might become apparent when more genomes are available but at present the neutral null hypothesis cannot be rejected. Moreover, we are a little suspicious of the selective hypothesis because, if anything, host-associated bacteria have slightly more TAS than terrestrial and multi-environmental organisms relative to the number expected from the size of their genomes (Table *4). *So there seems to be no trend in parasites and symbionts in general, an observation that suggests extra caution with regard to the selective hypothesis of TAS loss in intracellular microbes. Again, this hypothesis remains legitimate but so far no statistical evidence*.

8. We are not convinced of the biological meaning of the concept of "anti-addiction" by TA loci as described in Ref 25. Rather, we see the results therein as a mere consequence of how the experiments were set-up and any genuine biological meaning of anti-addiction remains to verified – that is – at its present experimental stage, it's simply too speculative as to incorporate into the already very long list of possible functions for TA loci. Rather, we should make an attempt to pin-point the facts known about TA loci thus to reduce the confusion in the field.

**Authors' response**: *We find the concept of anti-addiction very sensible and appealing. However, we do not attempt to carefully assess the validity and implications of the experiments described in ref*. [25], *so the presentation of this idea in the text is very cautious and framed with "could" and "might", and some more such qualifiers were added in the revision*.

### Reviewer 2

**Daniel Haft**, J. Craig Venter Institute

This work by Makarova, Wolf, and Koonin reports results from a comprehensive study of prokaryotic toxin-antitoxin system (TAS) protein pairs. These systems once were viewed simply as addiction modules that enforce plasmid maintenance by post-segragational killing upon plasmid loss. However, TAS gene pairs frequently contain a bacteriostatic rather than bacteriocidal toxin, occur chromosomally rather than on plasmids, and perform important regulatory functions in cellular responses to stress. Their detection, however, is tricky because of their small protein sizes, high diversity, and sparse experimental work. They are far more common than once thought, an average ten pairs per prokaryotic genome but sometimes much higher, and therefore are important to detect.

This work serves two functions. First, it is a broad, thorough, well-informed, hundred-plus reference review of the state of the art in predicting and interpreting TAS systems. The survey is essential to the bioinformatic analysis it enables. Second, it is a report of comprehensive predictive analysis of TAS systems in a collection of 750 prokaryotic reference genomes that features a number of new discoveries.

Distinct TAS systems that share no protein-level homology often show similarity in various other attributes: small protein size, arrangement in two-gene operons, sporadic distribution, absence of transmembrane domains from both, presence of a DNA-binding domain in one, and frequent association with plasmid and prophage regions. Several of these filtering criteria were combined in a computational pipeline complemented by human close review, the article's "method 1", which efficiently rediscovered a considerable number of known TAS systems and suggested a few others. The rediscovery helps validate the method, as do repeated demonstrations of appropriate remote sequence relationships in the new systems. The suggested new systems provide a rather large collection of bioinformatics-generated specific functional hypotheses for testing. The scope of this work is a reminder that comparative genomics still is underutilized as a discovery method for the preliminary characterization of largely novel biological systems.

A second computational approach used sensitive iterated searches to push the identification of known and hypothesized TAS pairs close to the limits of detection. This phase explored the notion that TAS modules do not always pair the same toxin family with the same antitoxin family, but rather can exchange families somewhat promiscuously. Again, filtering criteria and well-informed human review followed the computation, so the resulting proposed TAS pairs serve as excellent sets of hypotheses, suitable for greatly improving genomic annotation systems and spurring downsrteam studies. A number of follow-up questions spring to mind.

Type 2 TAS systems act as regulators of their own expression, but *are there other sites to which antitoxins or TA complexes would bind to regulate expression of other genes?*

**Authors' response**: *To our knowledge, no. We are unaware of any experimental evidence or computational study that would identify or predict potential "antitoxin regulons". Search for such regulons indeed would be interesting to pursue considering the fact that some of the antitoxin-binding sites are known, but this is definitely a separate analysis that is beyond the scope of this work*.

Do chromosomal TAS modules appear to act as carriers or guardians of neighboring "fitness factor" genes that ultimately benefit their host cells? What classes of cellular genes most commonly have TAS cassettes nearby?

**Authors' response**: *Very interesting questions indeed, we are currently investigating these issues*.

The limitations of this article for many scientists will lie not in the analyses themselves, but in achieving easy downstream use of the findings. The reported findings are extensive: an implicit biological database of curated TAS gene pairs with curated gene contexts, named protein families, well-researched sequence relationships, and putative functions. An important point is that the protein family definitions alone do not sufficiently represent all the work reported here – many proteins that belong to the families are unpaired orphans or otherwise out of context (see figure 11), and having the final sets of approved pairs itself is important. Therefore I would like to suggest that this paper be paired with supplemental or post-publication materials that explicitly provide the proteins themselves. A tab-delimited file might be sufficient, listing protein unique identifier, species, toxin/antitoxin protein family, COG family id (which I expect not to be exactly synonymous), and partner protein id. Such a resource would be immediately useful for genomic and metagenomic annotation pipelines and in spurring further studies as of DNA binding sites. Currently, varied nomenclatures in the TAS literature and other layers of indirection between publications and protein identifiers are hindering efficient community use of the synthesized knowledge reflected in this paper.

Either supplementary material as part of the publication, or a file deposited post-publication to the Readers' Comments section, of a database-like dump of the collected curated gene pairs would be invaluable.

**Authors' response**: *We agree and accordingly prepared the file with this information for individual toxin and antitoxin families, and for the TA pairs (see Additional File *14).

Method 1 finds a number of different previously known TAS systems, part of the proof of the validity of the method. But I did not find a statement about what fraction of previously known TAS systems was found and what known types were not found. I would expect the rather stringent requirement that at least one genome have at least three pairs of given type in order to nominate the type to cause some known types to be missed. In fact, I imagine there are some known TAS pairs where one or both lack a matching COG family. What TAS systems known to you before you started Method 1 were missed by its stringent filtering criteria?

**Authors' response**: *The questions about false positives and false negatives would be fully relevant if we proposed the two approaches used here to predict TAS (method 1 and method 2) as general methods for TAS prediction/identification. However, this is not the case. Rather, these approaches comprise data mining or "fishing expeditions" the goal of which is to predict widespread novel TAS that so far have not been discovered by experimental approaches. Therefore we used extensive manual curation in the course of all work and considered various additional lines of evidence*.

To me, one of the most intriguing partitions is that between chromosomal and plasmid positioning, broken down by family. Which of the TAS systems are usually plasmid, and which are usually chromosomal. Did you do this study?

**Authors' response**: *This is undoubtedly an interesting question. There are some problems with plasmid sequences and their identification that call for caution: it can be difficult to distinguish plasmid-derived regions on the chromosomes without much additional work; furthermore, the current plasmid databases are heavily biased toward plasmids of gamma proteobacteria and firmicutes, especially, industrially important ones, so it is not quite clear how to interpret the data. None of the known TAS occur exclusively on plasmids but there are a few that so far were found only on chromosomes. The cases when a particular TAS was often present on plasmids or phages are mentioned in the text. The new Table *6*shows the results of an additional statistical analysis that we undertook to address this question. Clearly, there are some TAS that prefer plasmids and others that prefer chromosomes*.

**Table 6:** Over-representation of TAS on plasmids and chromosome

**TAS**	**Number detected on plasmids**	**Number detected on chromosomes**	**Enrichment (p < 0.01)**
MazF-PHD	12	7	Plasmid

COG5654-Xre	55	195	Plasmid

MerR-PIN	9	34	Plasmid

GNAT-RHH	28	154	Plasmid

RelE-RHH	92	511	Plasmid

ArsR-COG3832	13	310	Chromosome

DUF397-Xre	3	129	Chromosome

HEPN-MNT	11	572	Chromosome

GNAT-Xre	0	67	Chromosome

The enrichment was estimated compared to the random expectation given the analyzed amount of chromosomal and plasmid sequences. The distributions of the rest of the TAS were statistically indistinguishable from the random expectation.

The model of chromosomal TAS as a means to prevent addiction to plasmid-mediated post-segragational killing is attractive. This kind of TAS cross-talk, and abundant TAS lateral transfer, suggests there should be a considerable number of dead TAS systems: silenced, truncated, point mutations, etc. Did you find evidence of these?

**Authors' response**: *A genuinely interesting question that is difficult to answer. The majority of the TA families are small, rapidly evolving proteins. Even toxin protein sequences (mostly enzymes) often do not have a single position that would be conserved throughout the entire family, suggesting the possibility that some of the toxins are inactivated (PINs are a good example). It is quite an intriguing possibility that some of these proteins might be functional toxins even in the absence of the enzymatic activity but this of course remains a speculation. Pseudogenes and truncations are harder to detect especially given the typical small size of the TA genes. What we know for a fact is that there are many solo toxins and antitoxins, and these can be reasonably viewed as derivatives of TAS that were exapted for other functions (Figure *11).

Your filtering criteria required both toxin and antitoxin to be within 100 bp of each other, but also that the pair not belong to a larger operon. It would be interesting to have a list of pairs that met the first criterion but were excluded through the second. These would be candidates for TAS systems being used in novel ways.

**Authors' response**: *This information is available in Table *2, *in the column "Adjacent gene function; reasons if discarded". There are some systems that potentially could be novel TAS, for instance, those that encode a membrane protein as a putative toxin. However, a detailed investigation of such cases is beyond the scope of the paper*.

I have read that TAS often flank pathogenicity islands (e.g. [107]), and have wondered about chromosomal TAS modules performing guard functions for neighboring genes. Have you explored these connections yet?

**Authors' response**: *This is a very interesting and potentially important issue that is the subject of our active, ongoing investigation*.

I saw a recent paper, January 2009, on connecting TAS to phage abortive infection proteins. And clearly, TAS mediated suicide as a means to protect clonally identical sister cells is a sensible mechanism for viral resistance. A small adjustment to the discussion, and one more reference, seems warranted.

**Authors' response**: *We are aware of this link and mention the parallel between the Abi and TA systems *[102]. *We also added a sentence on the direct mechanistic parallel demonstrated in the recent interesting paper from the Salmond lab *[103]. *Again, this connection is one of the central subjects of our ongoing, large-scale efforts on the characterization of the prokaryotic mobilome*.

### Reviewer 3

**Arcady R. Mushegian**, Stowers Institute for Medical Research and Kansas University Medical Center

This is a thorough computational study of toxin-antitoxin systems in prokaryotes. I have no qualms about sequence similarities and structure prediction, and only a few technical concerns, but I also feel that the broader biological context requires elaboration. More specifically:

1. Background: the explanation of the post-segregational killing mechanism (italics mine)

"The antitoxin is *metabolically unstable *whereas the toxin is stable. Therefore, unless the antitoxin is continuously replenished through gene expression, the free toxin accumulates in amounts sufficient to kill a cell, which is what occurs after cell division if a daughter cell does not receive the TAS-encoding plasmid"

seems to be at odds with the statement two paragraphs later:

"The antitoxin binding inhibits the activity of the toxin, and the *stable *TA complex binds to the operator of the corresponding TAS operon via the DNA-binding domain of the antitoxin and (auto)represses its transcription."

-- is antitoxin only unstable when not in complex with toxin, whereas the toxin-antitoxin complex is stable? Or is antitoxin also unstable when in complex (or perhaps the complex itself is unstable)? The kinetic aspect seems to be missing! Also, what is the molecular connection between toxin release from the pair and post-segregation?

**Authors' response**: *The first of the quoted statements was indeed incomplete in the original text. It is replaced with: "The antitoxin is metabolically unstable unless in a complex with the toxin, whereas the toxin is considerably more stable." We believe that this amendment should take care for any potential confusion. As for the "molecular connection between toxin release from the pair and post-segregation", the obvious connection is between the toxin release and post-segregational killing (not post-segregation per se): if one of the daughter cells has no means to produce the antitoxin, it is killed by the remaining toxin*.

2. Results and Discussion (pp 8–10) and correspond part of the Methods section: The approach that involves the analysis of the coefficient of variation of genes in COGs appears to have identified lots of TAS, but it is not quite clear why this approach should work with such specificity. At least 70% of all COGs have patchy phyletic distribution. Moreover, many COGs may be inherited in short operons, and, more specifically, such tandems as ATPase+permease subunits of transporters, or restriction+modification enzymes, or two-component signal transduction systems, are likely to belong to COGs with widely different paralog membership in different species. Have they been discovered by this approach? If not, why not? Perhaps the authors can discuss in more detail the properties of the whole distribution of COGs by CV values, in addition to its extreme, to mention the range of the CV values of the candidates discarded because they belonged to longer operons, etc.

**Authors' response**: *We expanded the Methods section, addressing some of these issues. The approach using the coefficient of variation is not extremely specific, so applied filtering that is described both in Figure *1*and in the Methods section. We also include Additional File *1*which is an annotated list of the 315 gene pairs that were obtained after automatic analysis of initial top 2000 COGs and were analyzed one by one using the STRING program (for all cases where the necessary information was present in the STRING system) or by manual neighborhood analysis in the few remaining cases. In this file, one can see which gene pairs other than TAS were analyzed. Among these were, of course, many transporter subunits, two-component signal transduction systems, transposons, and other usual suspects. However, it is easy to see that the majority of these genes do not form stable two-gene operons, with a few exceptions such as transposons that again can be readily distinguished from TAS*.

"For such systems [i.e., "resistome" as defined by the authors], the boundary between selfishness and "normal" cellular function seems to be fuzzy and more a matter of convention than a real distinction."

If this is so, what is a reason to distinguish resistome from the rest of the mobilome? After all, any biosynthetic operon that is mobile may be "selfish" in the sense of J. Lawrence (i.e., it best provides for its own survival when transferred as a complete group of genes coding for a coherent module), but it also has "normal" cellular function. Moreover, the function of a considerable portion of TAS is not (or not only) resistance.

**Authors' response**: *Of course, these boundaries are intrinsically fuzzy. Nevertheless, we believe that the components of the resistome do show a characteristic balance between selfishness and "normal" functioning that distinguishes them, on one end of the mobility range, from transposons and other "genuine" mobile elements, and on the other end, from regular biosynthetic operons. The latter, probably, should not be considered bona fide members of the mobilome although any operon indeed shows a degree of selfishness *sensu *Lawrence, so that there is not sharp boundary between the mobilome and the rest of the prokaryotic gene pool as noted here and elsewhere (e.g*. [89]). *Objective delineation of the mobilome is a challenging task, a major problem we are currently working on*.

Two general questions:

A. Per the "compromise" proposal and other observations: does it follow that antitoxin genes may be distributed broader than the toxin genes (also because antitoxins may have multiple functions and perhaps multiple stabilizing partners)? Is this actually observed, or is there perhaps a wash with some antitoxins represented by small RNAs?

"A potential compromise between a purely selfish life style of TAS and integral cellular functions could be a role of chromosomally encoded TAS in the protection of prokaryotic cells against post-segregational killing induced by plasmid-encoded homologous TAS whereby the antitoxin encoded by a chromosomal gene sequesters a plasmid-encoded toxin. Experimental evidence of such protection was reported, and elimination of the chromosomal TAS in the presence of the respective plasmid did adversely affect the fitness of the host bacterium [25]."

**Authors' response**: *We do find many more antitoxins than toxins (see Figure *11) *but the contribution of other functions of the antitoxins is hard to assess specifically. It seems unlikely that the small RNA antitoxin substantially contribute to this bias. So far this class of antitoxins seems to be associated primarily with type I toxins that are not considered in this work*.

B. Toxins appear to be similar to bacteriophages in many regards (highly mobile; potentially toxic; in love-hate relationship with cells), an indeed some TAS are encoded by phage genomes.

What is the connection of TAS to CRISPR loci? Are there any short toxin-derived sequences in CRISPRs?

**Authors' response**: *There is no particularly strong link. Some TAS occur in the vicinity of CRISPR loci but so do many other genes that might belong to the broadly defined mobilome*.

Is there any correlation between the number of both types of loci in different genomes?

**Authors' response**: *No correlation beyond what is expected from the scaling of genes in a particular category with the genome size*.

Minor comments:

"Resistome domain of the prokaryotic mobilome": do we really speak like this?

**Authors' response**: *yes, we (the authors) see nothing wrong about it and so do speak like this. The reader is informed by this comment that others do not*.

"The "selfish altruism", or "responsible selfishness", of TAS-like systems..." Is this Chernyshevsky-Randian property of TAS-like systems a defining feature of the whole mobilome, or of its subset resistome?

**Authors' response**: *We appreciate this point and the fully appropriate references to Chernyshevsky (more or less, intended when coining the terms) and Ayn Rand. Upon consideration, we indeed replaced "mobilome" with "resistome" as some other members of the mobilome might show less restrain and responsibility*.

The first sentence of Conclusions. Replace "We report here the most detailed and comprehensive comparative-genomic analysis of type II TAS so far available (to our knowledge)" by "We report here a comparative-genomic analysis of type II TAS"?

**Authors' response**: *We understand that this could read like honking our horn. However, the phrase carries a message. We kept it*.

### Reviewer 4

**Andrei Osterman**, The Burnham Institute

The excellent article by Makarova, Wolf and Koonin could be indeed termed Genomic Encyclopedia of Prokaryotic TAS-2 for the breadth and depth of coverage of this fascinating system over 750 prokaryotic genomes. However, the accurate cross-genomic projection of the presently known TAS components, no matter how challenging and important, is only one of the remarkable accomplishments of this study. By pushing the boundaries of predictive comparative genomics to a completely new level, the authors also substantially expanded a repertoire of known toxins and antitoxins. They developed a sophisticated workflow elegantly capturing the elusive and mysterious nature of TAS operons, which allowed them to predict dozens of previously unknown players and combinations thereof. This workflow (computational techniques and filters included therein) is yet another remarkable deliverable of this study, which will be inspirational and instructive for those who are chasing after other challenging protein families. The detailed analysis of the resulting monumental picture, a genomic distribution of thousands of TAS elements, provided new insights into their evolution, intra- and interspecies mobility and functional associations. No doubt, this single study provided a great starting point for dozens of experimental biologists to follow upon.

## Supplementary Material

Additional file 1 **The list of 315 adjacent pairs of COGs**. This file contains the list of 315 adjacent pairs of COGs that were further analyzed on a case by case basis for prediction of TAS (approach 1) as described in the Methods section. Click here for file

Additional file 2 **Multiple alignment of representative sequences of COG5606**. The alignment supports the analysis and description of the predicted *RelE-COG5606 *TAS Click here for file

Additional file 3 **Multiple alignment of truncated members of the MerR-like family**. The alignment supports the analysis and description of the predicted PIN-*MerR *TAS Click here for file

Additional file 4 **Multiple alignment of representative sequences of the XF1663 family**. The alignment supports the analysis and description of the predicted MazF-XF1663 TAS Click here for file

Additional file 5 **Multiple alignment of representative sequences of the YhfG family**. The alignment supports the analysis and description of the predicted YhfG-Fic/Doc TAS Click here for file

Additional file 6 **Multiple alignment of representative sequences of the DUF397 family**. The alignment supports the analysis and description of the predicted DUF397-HTH TAS Click here for file

Additional file 7 **Multiple alignment of representative sequences of the YgiU family**. The alignment supports the analysis and description of the predicted YgiU-xre TAS Click here for file

Additional file 8 **Multiple alignment of representative sequences of COG3832**. The alignment supports the analysis and description of the predicted COG3832-ArsR TAS Click here for file

Additional file 9 **Multiple alignment of a representative set of GNAT family acetyltransferases**. The alignment supports the analysis and description of the GNAT-xre and GNAT-RHH systems Click here for file

Additional file 10 **The representation of toxins, antitoxins and toxin-antitoxin pairs in 750 complete genomes of prokaryotes**. This file contains the counts of solo toxin and antitoxin genes as well as TA-pairs for all 750 prokaryotic genomes analyzed in this work. Phenotypic characteristics and the total number of protein coding genes are given for each genome. Click here for file

Additional file 11 **Statistical Appendix for the Methods**. The appendix contains additional details concerning the following procedures: i) the likelihood model used to estimate the scaling parameters for the number of TAS pairs relative to the genome size and ii) variability of abundance of different TAS pairs within ATGCs. Click here for file

Additional file 12 **Table S1**. Distribution of TAS among prokaryotic taxa Click here for file

Additional file 13 **Species abbreviations used in the alignments in Figures **2, 4, 5, **and **6. The provided table spells out the species abbreviations used in the alignments in Figures 2,4,5 and 6 Click here for file

Additional file 14 **Complete list of GenBank identifiers for all predicted and known toxins and antitoxins**. The table contains the GenBank identifiers (GI numbers) for all predicted and known toxins and antitoxins identified in this work in 750 complete prokaryotic genomes Click here for file
