# Supplementary material for: Comprehensive comparative-genomic analysis of Type 2 toxin-antitoxin systems and related mobile stress response systems in prokaryotes
Source: Biol Direct. 2009 Jun 3;4:19. doi: 10.1186/1745-6150-4-19 (PMC2701414; doi:10.1186/1745-6150-4-19)
Supplement: Additional file 4 — Multiple alignment of representative sequences of the XF1663 family. The alignment supports the analysis and description of the predicted MazF-XF1663 TAS [file 1745-6150-4-19-S4.doc]

85373510 Erylito 6 PLTDRQRVARHRAKLRAQ**G**LR**P**KQF**W**LP-DLGDPRIRSEIAGQCRKVSQ-HAETAEAQDLIDAI 67

39934019 Rhopalu 24 RSASAERVRRHREKMKAM**G**LK**P**VTI**W**VPEDYNSPEYKAEIRRECELINA-DPESEIVLEGMWEL 86

111025298 Rhosp- 1 MASTRERVREHRRRLREQ**G**LR**P**VQI**W**VP-DVRAPEFVAEAHRQSAAVAA-SEHEADDQAFVDAI 62

118469107 Mycsmeg 1 MTPARDRVRRHRERLRRQ**G**LR**P**VQI**W**VP-DVNAPEFRREAHRQSELVAA-GEHEAEDQAFVDAI 62

148556289 Sphwitt 7 PKPVRVKVREHRERLRAL**G**LR**P**IQI**W**VP-DVRSPSFRDQARRQSQAVAA-SAHAAEDQAFIDAV 68

78045268 Xancamp 2 SSIHESKVARHRERMRAA**G**LR**P**VQF**W**VP-DTRSPEFAAQVRKQCQNLKG-DPAETEVLRFTEEA 63

15838461 Xylfast 4 THRPDSKIVHHREQMRAA**G**LR**P**VQL**W**VP-DTRTPEFAAEIQSQCRALKG-DQAEADALRFTEKA 65

107021869 Burceno 2 AATTSERVRIHRENLRAA**G**LR**P**IQI**W**VP-DVRRPGFADECARQSRMVHQ-SIDENDLLDFIEQA 63

30249286 Niteuro 39 TTHVNARVKKHRDTLRMA**G**LR**P**VQI**W**VP-DTRRPDFAEECRRQCLLIAQADKADTSMQQFMDEA 101

56477328 Azosp- 3 NTPVNARVQKHRDARRRA**G**LR**P**VQI**W**VP-DTRRPDFAEECRRQSRLAAEADMADTDMQHFMDEA 65

21264261 Xanaxon 1 MAHVNSRVQKHRDALRMA**G**LR**P**VQI**W**VP-DTRRPDFAEECRRQCRLAAQADMADTDMQRFMDEA 63

78188801 Chlchlo 1 MVDVVARVHKHRVKLRDE**G**MR**P**IQL**W**VL-DTRREGFAEECHRQSALLAN-DSHEDEMMLFLSEV 62

67158996 Azovine 32 MQTVRERVRKRREALRAA**G**LR**P**VQI**W**VP-DTRAPGFAEEARRQCLLVQN-DPQEKESLEWLESA 93

78187760 Pellute 16 MAGVAERVQKYRAGLRKA**G**MR**P**LQI**W**VP-DTRRAGFAAECRRQSALLKK-DHQEKEVLQFLEKA 77

119357200 Chlphae 1 MKVVTERVKRYRDKLRER**G**LR**P**VQI**W**VA-DTRRPGFELECRRQSALLKS-DAHEKEVLEFLERA 62

PSIPRED --HHHHHHHHHHHHHHH------EEE-------HHHHHHHHHHHHHHH---HHHHHHHHHHHHA

consensus/100% ......ph..hR..hp..GhpPhphWh..s.....h..p.......h................h

**XF1863 family**
