# Supplementary material for: Comprehensive comparative-genomic analysis of Type 2 toxin-antitoxin systems and related mobile stress response systems in prokaryotes
Source: Biol Direct. 2009 Jun 3;4:19. doi: 10.1186/1745-6150-4-19 (PMC2701414; doi:10.1186/1745-6150-4-19)
Supplement: Additional file 6 — Multiple alignment of representative sequences of the DUF397 family. The alignment supports the analysis and description of the predicted DUF397-HTH TAS [file 1745-6150-4-19-S6.doc]

21224455 Strcoel 21 CIEVAYE**W**FKSSYS-GDEGGN**C**VEVAQEP-T--AVHI**R**DSKAPQG-HVTVGPDAWAAFL-GAR 77

21224070 BldB 23 LDISGVE**W**HSAPGT--EEHEERVEIAYLP-DG-AVAM**R**SSLDPE-TVLRYTEAEWRAFVLGAR 80

56707102 Stralbu 10 ALLAGAQ**W**FKSEAS--SASGG**C**LEVAFLP-GD-LVGIRDNEDLTNPPFVVTKHVWNCWVAGAK 68

125634470 Amybenz 7 TPPAGLV**W**RKARAS--QGDND**C**VYVADDP-GN-GGRWLDESATDQPPSFVPESSWQAFLTGAK 65

145594135 Saltrop 3 ESLPEVA**W**HISTKS-DSNGGS**C**VEAGPLLDGSGRVAV**R**HSKAPEAATIIYTAEEWAAFVGGVR 64

29831742 Straver 7 KVDDSSM**W**FTSSYS-NGAGGE**C**VECASSD-D--NALI**R**DSKRLGGPVVGVPGLAWHAFVRALK 65

85813867 Strrimo 11 SAIEGAV**W**RKSSRS--NPNGN**C**VELAVLA-DG-GVAV**R**NSRHTGGPALVYTRDEIAAFVQGAK 69

134103171 Saceryt 1 --MTEPK**W**RISSYS---QGHE**C**VETAVLP-E--VTLV**R**DSKAPDAGHFAVSAVRWRSFLSRVK 55

72160495 Thefusc 4 PDPAPLA**F**RKSSYS--VTAQE**C**VEVAVTS-E--FVAV**R**DSAHRELGYLTFPLAEWRAFLTELT 61

145596255 Saltrop 12 PDLARAA**W**RKSTRS---QTSN**C**VEVAPLP-A--TVAL**R**DSKDRGGPVLLFDRGEWRNFLAAAK 68

72162201 Thefusc 1 MHSPNVQ**W**RKSSYS--GSGDN**C**VEVAETP-EA-VVFV**R**DTQNRHLGYLKFSAQEWTAFLHTLK 59

29831837 Straver 8 NAATLRG**W**RKSSYS-NSEGGS**C**LEVLDGH-PS-GVPV**R**DSKVPHGPALVFSPADWASFVTAVK 67

21222693 Strcoel 21 NCLEVAR**W**RKSTHS-GGSGGN**C**LEVATGN-T--AVPV**R**DSKNPVGPTLRFRAEAWSVFVDGIR 79

145593684 Saltrop 2 NDRQLGP**W**RKSTRS--GGADN**C**VEVTTAT-DL-YVGV**R**DSKNPDG-VLVFGPDGWFEFIEGVR 59

54022458 Nocfarc 3 ADLSGAH**W**FKSTFS--NAGGE**C**VEIAHMD-NG-GVRV**R**DSKNPSGAVLTFTPAEWDAFLAGVR 61

86741393 Frasp 4 TDLTNAE**W**RKSSYS--ATQTT**C**VEVAQVD-E--VVAV**R**DSKDPTGPVLVFTPDEWAAFLAGVR 61

145594064 Saltrop 4 MDMTCVT**W**RKSTRS--NGSGN**C**VEVATEL-AG-EVGL**R**DSKDPSGPILTFSPTAWATFVRATQ 62

134098131 Saceryt 1 MDLADAH**W**RKSSRTGVGGNGN**C**VEVAFVG-A--AVAV**R**DSKDPDGAALAFTHEAWAAFLDRLS 60

145592669 Saltrop 1 MDLTGAR**W**RTSSRS-SGNGGD**C**VEVADNL-PG-VVGV**R**DSKDPTGPVLVFGPASWGAFVAQLP 60

29830592 Straver 8 PDLTTAV**W**RKSSYS-DGGDSN**C**VEVADGY-PG-LVPV**R**DSKAPQGPALVFGAEPWAAFLAMAK 67

PSIPRED ------EEEE-----------EEEEE-------EEEEE--------EEEE-HHHHHHHHHHHH

consensus/95% .......W.ps..o.......Clph.........h.hRpop......h.hs...h..hh..h.

**DUF397 family**
