# Supplementary material for: Comprehensive comparative-genomic analysis of Type 2 toxin-antitoxin systems and related mobile stress response systems in prokaryotes
Source: Biol Direct. 2009 Jun 3;4:19. doi: 10.1186/1745-6150-4-19 (PMC2701414; doi:10.1186/1745-6150-4-19)
Supplement: Additional file 11 — Statistical Appendix for the Methods. The appendix contains additional details concerning the following procedures: i) the likelihood model used to estimate the scaling parameters for the number of TAS pairs relative to the genome size and ii) variability of abundance of different TAS pairs within ATGCs. [file 1745-6150-4-19-S11.doc]

***Estimate of the scaling parameters.***

Let us assume the following model of relationship between the genome size and the number of genes belonging to a particular class (e.g. TA gene pairs): for a given genome size *l* the expected number of genes is given by *m* = *bl^a^* (allometric scaling). Additionally, assume that the logarithm of the observed number of genes tends to be normally distributed with the mean ln(*m*) = *a*ln(*l*)+ln(*b*) (which depends on *l*) and standard deviation *s* (independent of *l*). Since the number of genes in a genome is by definition integer, expected values <<1 translate to observed values of 0.

Formally, let us assume that the integer number of observed genes *k* corresponds to a real number interval of [*k*‑0.5,*k*+0.5], except for *k*=0 which corresponds to [0,0.5]. Then the probability to observe *k* genes in the genome of size *l* is given by

$P\{k\}=\left\{\begin{matrix} N\left(\ln(k+0.5),\ln(m),s\right)-N\left(\ln(k-0.5),\ln(m),s\right) & ,k>0 \\ N\left(\ln(0.5),\ln(m),s\right)-N\left(-\infty,\ln(m),s\right) & ,k=0 \end{matrix}\right.$ (1)

where *N*(*x*,*y*,*z*) is a cumulative normal distribution function at point *x* with mean *y* and standard deviation *z* and *m* = *bl^a^*.

For a given set of genomes the log-likelihood of observing the particular vector of the number of genes is

$L=\sum_{i=1}^{n} \ln(P\{k_{i}\})$ (2)

where *P*{*k_i_*} is given by eq. (1). Given the data, *L* depends on the model parameters *a*, *b* and *s*; these parameters can be adjusted to produce the maximum likelihood value, providing the optimum scaling factors *a* and *b* and the spread factor *s*.

For the TAS genes observed in 750 completely sequenced prokaryotic genomes the maximum likelihood model is defined by the allometric scaling factors *a* = 1.64 and *b* = 8.3x10^‑6^ (see Figure A1, solid red line) and the spread factor *s* = 1.12.

Approximate boundaries of the 95% confidence intervals for the number of observed genes are given by

*be^±^*^1.96^*^s^l^a^* (3)

(see Figure A1, dashed red lines). The largest genome size for which observation of zero genes is still likely (i.e. within the 95% confidence interval) can be estimated as the solution of the lower-boundary version of eq. (3) for the value of 0.5, or *e*^(ln(0.5)+1.96^*^s^*^‑ln(^*^b^*))/*^a^*. For the TAS genes this corresponds to the genome size of 3088 protein-coding genes. The largest genome with no TAS pairs detected, *Prochlorococcus marinus* MIT 9303, has 2997 genes, a lower (albeit remarkably close) number. Thus we can conclude that the absence of TAS gene pairs in certain genomes fits well with the expectations of the allometric scaling model and does not require additional explanations.

**Figure A1.**

***Variability of abundance of different TAS pairs within ATGCs.***

We examined the distribution of TAS in the 41 set of closely related prokaryotic genomes (Alignable Tight Genomic Clusters, ATGC [1, 2]). In 33 of the ATGC at least one of the 37 TAS was detected in at least one of the members. Additionally, all proteins of the 163 ATGC members were assigned to COGs [3].

For each ATGC and each TAS pair the standard deviation of the number of the given TAS pairs in each of the genomes of the given ATGC was computed if this TAS pair was present in at least one genome of this ATGC. Average of these values was computed across the TAS pairs within each ATGC; the results are reported in the table below (Table A1, “TAS” column).

As a control, a random sample of 37 COGs with consensus protein length less or equal to 150 a.a. was selected; the standard deviations of the number of proteins for each COG and each ATGC were computed in the similar manner; the average values are reported in the table below (“COG” column).

Overall, for 32 out of 33 ATGCs TAS pairs display higher variability of the per-genome numbers than that of COGs. Hypothesis of the equal variability of TAS and COGs is rejected with p-value of 4x10^‑9^ (exact binomial test with *n* = 33 and *p* = 0.5).

1. Novichkov PS, Ratnere I, Wolf YI, Koonin EV, Dubchak I: **ATGC: a database of orthologous genes from closely related prokaryotic genomes and a research platform for microevolution of prokaryotes**. *Nucleic Acids Res* 2008.

2. Novichkov PS, Wolf YI, Dubchak I, Koonin EV: **Trends in prokaryotic evolution revealed by comparison of closely related bacterial and archaeal genomes**. *J Bacteriol* 2008.

3. Tatusov RL, Fedorova ND, Jackson JD, Jacobs AR, Kiryutin B, Koonin EV, Krylov DM, Mazumder R, Mekhedov SL, Nikolskaya AN *et al*: **The COG database: an updated version includes eukaryotes**. *BMC Bioinformatics* 2003, **4**:41.

**Table A1.**

| ATGC | no. of genomes | TAS | COG |
| --- | --- | --- | --- |
| *Aeromonas sp.* | 2 | 0.71 | 0.38 |
| *Anabaena sp.* | 2 | 0.93 | 0.24 |
| *Bradyrhizobium sp.* | 2 | 0.81 | 0.47 |
| *Geobacillus sp.* | 2 | 0.61 | 0.49 |
| *Nitrobacter sp.* | 2 | 0.86 | 0.63 |
| *Psychrobacter sp.* | 2 | 0.71 | 0.45 |
| *Rhizobium sp.* | 2 | 1.46 | 0.33 |
| *Rhodopseudomonas palustris* | 2 | 0.71 | 0.27 |
| *Roseiflexus sp.* | 2 | 0.57 | 0.35 |
| *Salinispora sp.* | 2 | 0.86 | 0.47 |
| *Sinorhizobium sp.* | 2 | 1.26 | 0.41 |
| *Thermotoga sp.* | 2 | 0.71 | 0.20 |
| *Xylella fastidiosa* | 2 | 0.59 | 0.00 |
| *Dehalococcoides sp.* | 3 | 0.58 | 0.41 |
| *Lactococcus lactis* | 3 | 0.72 | 0.23 |
| *Methanococcus maripaludis* | 3 | 0.53 | 0.14 |
| *Pseudomonas aeruginosa* | 3 | 0.36 | 0.12 |
| *Pseudomonas sp.* | 3 | 0.73 | 0.22 |
| *Pseudomonas syringae* | 3 | 0.80 | 0.29 |
| *Rhodobacter sphaeroides* | 3 | 0.83 | 0.40 |
| *Helicobacter sp.* | 4 | 0.58 | 0.10 |
| *Listeria sp.* | 4 | 1.00 | 0.18 |
| *Neisseria sp.* | 4 | 0.53 | 0.24 |
| *Burkholderia sp.* #1 | 5 | 0.92 | 0.43 |
| *Campylobacter jejuni* | 5 | 0.67 | 0.13 |
| *Brucella sp.* | 6 | 0.61 | 0.38 |
| *Rickettsia sp.* | 6 | 1.10 | 0.59 |
| *Xanthomonas sp.* | 6 | 0.56 | 0.28 |
| *Shewanella sp.* | 8 | 0.82 | 0.38 |
| *Bacillus sp.* | 9 | 0.31 | 0.67 |
| *Burkholderia sp.* #2 | 9 | 0.48 | 0.28 |
| *Yersinia sp.* | 9 | 0.59 | 0.22 |
| *Escherichia coli* | 21 | 0.68 | 0.59 |

List of COGs randomly selected for comparison: COG0695, COG0724, COG0831, COG1145, COG1310, COG1324, COG1334, COG1369, COG1558, COG1695, COG1708, COG1937, COG2051, COG2075, COG2402, COG2827, COG2841, COG3439, COG3502, COG3755, COG3782, COG3860, COG3951, COG4095, COG4654, COG4710, COG4852, COG4933, COG4954, COG4999, COG5302, COG5417, COG5469, COG5531, COG5586, COG5614, COG5634.
