# Supplementary material for: Comprehensive comparative-genomic analysis of Type 2 toxin-antitoxin systems and related mobile stress response systems in prokaryotes
Source: Biol Direct. 2009 Jun 3;4:19. doi: 10.1186/1745-6150-4-19 (PMC2701414; doi:10.1186/1745-6150-4-19)
Supplement: Additional file 12 — Table S1. Distribution of TAS among prokaryotic taxa [file 1745-6150-4-19-S12.doc]

Table 4. Distribution of TAS among prokaryotic taxa

| TA pair | Crenarchaeota | Euryarchaeota | Korarchaeota | Nanoarchaeota | Actinobacteria | Aquificae | Bacteroidetes/Chlorobi | Chlamydiae | Verrucomicrobia | Chloroflexi | Cyanobacteria | Deinococcus-Thermus | Fibrobacteres/Acidobacteria | Bacilli | Clostridia | Mollicutes | Fusobacteria | Planctomycetes | Alphaproteobacteria | Betaproteobacteria | delta/epsilon proteobacteria | Gammaproteobacteria | Spirochaetes | Thermotogae |
| --- | --- | --- | --- | --- | --- | --- | --- | --- | --- | --- | --- | --- | --- | --- | --- | --- | --- | --- | --- | --- | --- | --- | --- | --- |
| AbrB/Fic | 0 | 0 | 0 | 0 | 0 | 0 | 9 | 2 | 0 | 0 | 3 | 0 | 2 | 9 | 3 | 0 | 0 | 0 | 27 | 5 | 2 | 5 | 2 | 0 |
| AbrB/MazF | 0 | 1 | 0 | 0 | 0 | 0 | 3 | 0 | 0 | 1 | 5 | 2 | 2 | 12 | 2 | 0 | 0 | 0 | 12 | 25 | 7 | 40 | 6 | 0 |
| AbrB/PIN | 49 | 57 | 5 | 0 | 17 | 0 | 20 | 0 | 0 | 1 | 15 | 5 | 1 | 1 | 25 | 0 | 0 | 0 | 83 | 52 | 14 | 78 | 7 | 0 |
| AbrB/RelE | 0 | 3 | 0 | 0 | 0 | 0 | 0 | 0 | 0 | 0 | 2 | 0 | 1 | 2 | 6 | 0 | 0 | 0 | 9 | 10 | 0 | 16 | 0 | 0 |
| ArsR/  COG3832 | 0 | 0 | 0 | 0 | 86 | 0 | 6 | 0 | 1 | 1 | 3 | 0 | 11 | 18 | 1 | 0 | 0 | 0 | 100 | 49 | 24 | 9 | 14 | 0 |
| Bro/Xre | 0 | 0 | 0 | 0 | 0 | 0 | 0 | 0 | 0 | 0 | 0 | 0 | 0 | 43 | 12 | 0 | 0 | 0 | 2 | 0 | 0 | 1 | 0 | 0 |
| COG2442/PIN | 2 | 3 | 0 | 0 | 13 | 0 | 6 | 0 | 0 | 8 | 57 | 0 | 6 | 0 | 3 | 0 | 0 | 0 | 15 | 3 | 1 | 2 | 0 | 0 |
| COG2856/Xre | 0 | 1 | 0 | 0 | 18 | 0 | 0 | 0 | 0 | 3 | 6 | 0 | 2 | 114 | 40 | 0 | 1 | 1 | 27 | 21 | 3 | 11 | 0 | 0 |
| COG2886/PIN | 10 | 17 | 1 | 0 | 0 | 0 | 5 | 0 | 0 | 3 | 19 | 0 | 0 | 1 | 3 | 0 | 0 | 0 | 1 | 0 | 2 | 0 | 2 | 0 |
| COG2886/RelE | 0 | 0 | 0 | 0 | 0 | 0 | 0 | 0 | 0 | 0 | 2 | 0 | 0 | 0 | 0 | 0 | 0 | 0 | 0 | 0 | 0 | 0 | 0 | 0 |
| COG2929/RHH | 0 | 0 | 0 | 0 | 2 | 0 | 16 | 0 | 0 | 1 | 14 | 2 | 2 | 0 | 1 | 0 | 0 | 0 | 51 | 30 | 18 | 55 | 9 | 0 |
| COG2929/Xre | 0 | 0 | 0 | 0 | 0 | 0 | 0 | 0 | 0 | 0 | 0 | 0 | 0 | 0 | 0 | 0 | 0 | 0 | 4 | 0 | 1 | 0 | 0 | 0 |
| COG3832/Xre | 0 | 0 | 0 | 0 | 0 | 0 | 0 | 0 | 0 | 0 | 0 | 0 | 0 | 0 | 0 | 0 | 0 | 0 | 4 | 1 | 0 | 0 | 0 | 0 |
| COG5606/RelE | 0 | 0 | 0 | 0 | 1 | 0 | 0 | 0 | 0 | 0 | 6 | 0 | 3 | 0 | 0 | 0 | 0 | 0 | 7 | 13 | 1 | 31 | 0 | 0 |
| COG5654/Xre | 0 | 0 | 0 | 0 | 21 | 0 | 9 | 0 | 0 | 0 | 7 | 0 | 1 | 0 | 0 | 0 | 0 | 0 | 75 | 72 | 8 | 57 | 0 | 0 |
| DUF397/Xre | 0 | 0 | 0 | 0 | 132 | 0 | 0 | 0 | 0 | 0 | 0 | 0 | 0 | 0 | 0 | 0 | 0 | 0 | 0 | 0 | 0 | 0 | 0 | 0 |
| Fic/PHD | 0 | 0 | 0 | 0 | 0 | 0 | 0 | 0 | 0 | 0 | 0 | 0 | 0 | 1 | 2 | 0 | 0 | 0 | 1 | 0 | 0 | 13 | 0 | 0 |
| Fic/RHH | 0 | 0 | 0 | 0 | 16 | 0 | 0 | 0 | 0 | 1 | 2 | 0 | 0 | 0 | 0 | 1 | 0 | 0 | 3 | 0 | 0 | 1 | 0 | 0 |
| Fic/Xre | 0 | 0 | 0 | 0 | 0 | 0 | 4 | 0 | 0 | 0 | 0 | 0 | 0 | 2 | 3 | 0 | 0 | 0 | 5 | 1 | 7 | 19 | 0 | 0 |
| Fic/YhfG | 0 | 0 | 0 | 0 | 0 | 0 | 0 | 0 | 0 | 0 | 0 | 0 | 0 | 0 | 0 | 0 | 0 | 0 | 0 | 0 | 0 | 30 | 0 | 0 |
| GNAT/RHH | 0 | 3 | 0 | 0 | 12 | 0 | 6 | 0 | 0 | 0 | 6 | 1 | 2 | 1 | 0 | 0 | 0 | 0 | 25 | 17 | 4 | 105 | 0 | 0 |
| GNAT/Xre | 0 | 0 | 0 | 0 | 2 | 0 | 5 | 0 | 0 | 0 | 0 | 0 | 0 | 3 | 2 | 0 | 0 | 0 | 11 | 29 | 0 | 15 | 0 | 0 |
| HEPN/MNT | 103 | 92 | 3 | 0 | 8 | 5 | 32 | 0 | 2 | 54 | 36 | 17 | 2 | 6 | 81 | 0 | 0 | 0 | 32 | 24 | 38 | 30 | 8 | 9 |
| HicA/HicB | 0 | 32 | 0 | 0 | 8 | 0 | 7 | 0 | 0 | 2 | 51 | 5 | 5 | 12 | 27 | 0 | 0 | 0 | 29 | 11 | 21 | 47 | 6 | 4 |
| HipA/Xre | 0 | 0 | 0 | 0 | 18 | 1 | 29 | 0 | 0 | 0 | 0 | 0 | 4 | 0 | 0 | 0 | 1 | 0 | 41 | 87 | 18 | 135 | 1 | 0 |
| MazF/PHD | 0 | 0 | 0 | 0 | 0 | 0 | 0 | 0 | 0 | 0 | 0 | 0 | 0 | 0 | 0 | 0 | 0 | 0 | 19 | 0 | 0 | 0 | 0 | 0 |
| MazF/RHH | 0 | 1 | 0 | 0 | 27 | 0 | 5 | 0 | 0 | 0 | 6 | 0 | 0 | 49 | 30 | 0 | 0 | 0 | 16 | 5 | 8 | 38 | 4 | 0 |
| MazF/XF1863 | 0 | 0 | 0 | 0 | 4 | 0 | 5 | 0 | 0 | 0 | 0 | 0 | 0 | 0 | 0 | 0 | 0 | 0 | 15 | 5 | 0 | 4 | 0 | 0 |
| MazF/Xre | 0 | 0 | 0 | 0 | 0 | 0 | 0 | 0 | 0 | 1 | 8 | 0 | 0 | 3 | 3 | 0 | 0 | 0 | 2 | 0 | 2 | 0 | 0 | 0 |
| MerR/PIN | 0 | 0 | 0 | 0 | 13 | 0 | 0 | 0 | 0 | 0 | 3 | 2 | 0 | 0 | 0 | 0 | 0 | 0 | 7 | 8 | 0 | 9 | 0 | 0 |
| PHD/PIN | 0 | 1 | 0 | 0 | 58 | 0 | 6 | 0 | 0 | 6 | 40 | 10 | 8 | 1 | 19 | 0 | 0 | 0 | 95 | 56 | 17 | 21 | 11 | 0 |
| PHD/RelE | 0 | 0 | 0 | 0 | 33 | 1 | 15 | 0 | 0 | 1 | 33 | 1 | 1 | 34 | 18 | 0 | 0 | 0 | 28 | 32 | 27 | 140 | 0 | 0 |
| PIN/RHH | 48 | 47 | 3 | 0 | 196 | 0 | 17 | 0 | 0 | 2 | 48 | 8 | 8 | 0 | 14 | 0 | 0 | 0 | 94 | 56 | 13 | 33 | 23 | 1 |
| PIN/Xre | 0 | 4 | 0 | 0 | 1 | 1 | 8 | 0 | 0 | 3 | 20 | 0 | 0 | 2 | 2 | 0 | 0 | 0 | 1 | 9 | 0 | 6 | 0 | 1 |
| RHH/RelE | 0 | 38 | 0 | 0 | 7 | 0 | 37 | 2 | 0 | 0 | 16 | 1 | 5 | 58 | 6 | 0 | 3 | 1 | 136 | 58 | 25 | 200 | 6 | 0 |
| RelE/Xre | 0 | 0 | 0 | 0 | 30 | 0 | 59 | 3 | 0 | 1 | 43 | 0 | 5 | 10 | 8 | 0 | 1 | 0 | 134 | 118 | 44 | 295 | 14 | 0 |
| Xre/YgiU | 0 | 0 | 0 | 0 | 0 | 0 | 5 | 0 | 0 | 0 | 0 | 0 | 1 | 5 | 5 | 0 | 0 | 0 | 2 | 9 | 4 | 22 | 0 | 0 |

For each division of Archaea and Bacteria, the number of instantiations of each TAS detected in all available genomes is indicated.
